# The RNA demethylase ALKBH5 promotes the progression and angiogenesis of lung cancer by regulating the stability of the LncRNA PVT1

**DOI:** 10.1186/s12935-022-02770-0

**Published:** 2022-11-15

**Authors:** Wenyi Shen, Juan Pu, Zhi Zuo, Shanye Gu, Jing Sun, Bing Tan, Lili Wang, Jianmeng Cheng, Yangsong Zuo

**Affiliations:** 1 Department of Respiratory Medicine, Lianshui County People’s Hospital, Kangda College of Nanjing Medical University, Huai’an, China; 2 Department of Radiotherapy, Lianshui County People’s Hospital, Kangda College of Nanjing Medical University, Huai’an, China; 3 Department of Cardiology, The First Affiliated Hospital of Nanjing Medical University, Nanjing, China

**Keywords:** Lung cancer, ALKBH5, Angiogenesis, PVT1, RNA stability

## Abstract

**Background:**

N6-methyladenosine (m6A) is the most common posttranscriptional modification of RNA and plays critical roles in human cancer progression. However, the biological function of m6A methylation requires further studied in cancer, especially in tumor angiogenesis.

**Methods:**

A public database was used to analyze the expression and overall survival of ALKBH5 and PVT1 in lung cancer patients. CCK-8 and colony formation assays were performed to detect cell proliferation, a transwell assay was used to assess cell migration, and a tube formation assay was performed to assess angiogenic potential in vitro. A zebrafish lung cancer xenograft model was used to verify the function of ALKBH5 and PVT1 in vivo. Western blot assays were used to measure the relative protein expression in lung cancer cells. SRAMP predictor analysis and RNA stability experiments were used to examine the potential m6A modification.

**Results:**

Bioinformatics analysis showed that the expression levels of m6A-related genes were changed significantly in lung cancer tissues compared with normal lung tissues. We then identified that ALKBH5 was upregulated in lung cancer tissues and associated with poor prognosis of lung cancer patients by analyzing a public database. Knockdown of ALKBH5 inhibited the proliferation and migration of cultured lung cancer cell lines. Zebrafish lung cancer xenografts showed that ALKBH5 silencing also suppressed the growth and metastasis of lung cancer cells. Moreover, knockdown of ALKBH5 inhibited the angiogenesis of lung cancer in vitro and in vivo. Mechanistic studies showed that knockdown of ALKBH5 decreased the expression and stability of PVT1 in lung cancer cells. We next observed that PVT1 promoted the progression of lung cancer cells in vitro and in vivo and regulated the expression of VEGFA and angiogenesis in lung cancer. Finally, rescue experiments revealed that ALKBH5 regulated the proliferation, migration and angiogenesis of lung cancer cells, partially through PVT1.

**Conclusion:**

Our results demonstrate that ALKBH5 promotes the progression and angiogenesis of lung cancer by regulating the expression and stability of PVT1, which provides a potential prognostic and therapeutic target for lung cancer patients.

**Supplementary Information:**

The online version contains supplementary material available at 10.1186/s12935-022-02770-0.

## Background

Lung cancer is one of the most common malignant tumors and the leading cause of human cancer deaths worldwide [1, 2]. Despite improvements in early detection, diagnosis, surgery and drug therapy, the 5-year survival rate of lung cancer patients is still low [3–5]. Notably, tumor metastasis, associated with poor prognosis, has been shown to occur in 60–70% of cancer patients [6]. Metastasis is a complex process that cancer cells spread from the tumor origin site to distant parts of the body and is the most common cause of cancer death in the world [6, 7]. Therefore, it is urgent to understand the molecular mechanisms of lung cancer metastasis in order to make an efficient diagnosis and therapeutic strategy and improve the survival rate of lung cancer patients.

N6-methyladenosine (m6A) is the most abundant internal modification of RNA in eukaryotic cells and has gained increasing attention in recent years [8–10]. This modification mainly affects protein expression at the posttranscriptional level through “writers”, “erasers” and “readers” and has been shown to play different biological roles by regulating RNA splicing, stability, degradation and translation [9, 11–15]. The m6A “writers” are m6A methyltransferases that add the methyl group to the m6A modification sites and include methyltransferase-like 3 (METTL3), methyltransferase-like 14 (METTL14), methyltransferase-like 16 (METTL16), and Wilms’ tumor 1-associated protein (WTAP) among others [16–19]. The m6A “erasers” are m6A demethylases that remove the methyl group from the m6A modification sites and include fat mass and obesity-associated protein (FTO) and α-ketoglutarate-dependent dioxygenase homolog 5 (ALKBH5) [20, 21]. The m6A “readers” recognize m6A-modified sites and include YTHDF1/2/3, YTHDC1/2, and IGF2BP1/2/3 among others [12, 15, 22]. Recently, increasing evidence has indicated that m6A methylation plays important roles in various diseases, such as hematopoietic diseases, central nervous diseases, reproductive system diseases, and human cancer [8, 15]. In human cancer, abnormal m6A modification has been reported to affect tumor proliferation, migration and invasion [8, 23]. ALKBH5, a demethylated enzyme, has been shown to participate in various biological processes of human cancer, such as growth and metastasis [23, 24]. In recent years, ALKBH5 has been shown to play oncogenic roles in lung cancer [25–27], but the mechanism still needs to be studied further.

Angiogenesis is the process by which new blood vessels form from preexisting vessels and plays important roles in normal growth, development, tissue regeneration and wound healing [28–31]. As one of the pivotal hallmarks in human cancer progression, angiogenesis has been reported to participate in tumorigenesis, especially metastasis [32–34]. Similar to normal tissues, tumor tissues need nutrients and oxygen to grow, which induces tumor-associated neovessels to sprout from the existing blood vessels and form the neovasculature toward the tumor to address these needs [34–36]. Under physiological conditions, angiogenesis is well regulated through an angiogenic switch. In tumor progression, the angiogenic switch is activated due to the disrupted balance between proangiogenic and antiangiogenic regulators [37–40]. Vascular endothelial growth factor A (VEGFA) is a well-known factor that plays critical roles in angiogenesis, and it is mainly released by tumor cells during the development of tumors [41, 42]. Recently, a few studies have indicated that m6A modification is involved in tumor angiogenesis. In colon cancer, the m6A reader IGF2BP3 can regulate angiogenesis [43]. In gastric cancer, METTL3 promotes angiogenesis by regulating the m6A modification of HDGF mRNA [44]. However, the role of ALKBH5 in tumor angiogenesis is still unclear.

In this study, we analyzed m6A modification-related genes in the TCGA database and Genotype-Tissue Expression (GTEx) database and found that the expression levels of these genes were changed in lung cancers. Here we chose ALKBH5 for this study. Bioinformatics analysis showed that ALKBH5 was upregulated in lung cancer tissues, and high expression levels of ALKBH5 were associated with poor prognosis in lung cancer patients. We observed that ALKBH5 promoted the proliferation and metastasis of lung cancer cells in vitro and in vivo. We also found that ALKBH5 regulated the angiogenesis of lung cancer in vitro and in vivo. Furthermore, the expression and stability of lncRNA PVT1 (long noncoding RNA plasmacytoma variant translocation 1) were regulated by ALKBH5, and PVT1 overexpression partially restored the proliferation, migration and angiogenesis of lung cancer cells, which was suppressed by ALKBH5 knockdown. Our results demonstrate that ALKBH5 contributes to proliferation, migration and angiogenesis through PVT1 in lung cancer, thus providing a potential antitumor therapeutic target for lung cancer patients.

## Materials and methods

### Bioinformatics analysis

We used the TCGA database and GTEx database to analyze the expression levels of the m6A modification-related genes in lung cancer tissues compared to normal tissues. The TNM plot website was used to analyze differential gene expression in various tumors (https://tnmplot.com/analysis/). The correlations were analyzed using Gene Expression Profiling Interactive Analysis (GEPIA; http://gepia.cancer-pku.cn/). Kaplan‒Meier analysis (https://kmplot.com/analysis/) and lnCAR software (https://lncar.renlab.org/) were used to analyze the overall survival (OS) of lung cancer patients. The SRAMP predictor was used to predict the potential m6A modification sites.

### Cell culture

The human lung cancer cell lines A549, H1299, H1975, and PC9 and the human bronchial epithelial cell 16HBE were obtained from the Institute of Biochemistry and Cell Biology of Chinese Academy of Science (Shanghai, China). The A549, H1975 and 16HBE cell lines were incubated in 1640 medium, and the PC9 and H1299 cell lines were maintained in DMEM. Both media were supplemented with 10% fetal bovine serum (FBS), 100 U/mL penicillin and 100 mg/mL streptomycin, and all cells were incubated in a humidified atmosphere with 5% CO2 at 37 °C.

### RNA extraction and qRT‒PCR

Total RNA was extracted from cultured cells using TRIzol reagent (Invitrogen, CA, USA). Then, reverse transcription to cDNA of the total RNA was performed using random primers according to the instructions of the PrimeScript RT kit (Takara, Dalian, China). The products were detected by quantitative real-time PCR (qRT‒PCR) using SYBR Green Master Mix according to the manufacturer’s instructions (Takara, Dalian, China). Glyceraldehyde 3-phosphate dehydrogenase (GAPDH) was used as an endogenous control, and the 2^−∆∆Ct^ method was used to analyze all data. The primer sequences for qRT‒PCR are listed in Table 1.

**Table 1:** Primer sequences for qRT‒PCR

Gene	Forward primer (5′–3′)	Reverse primer (5′–3′)
GAPDH	GGGAGCCAAAAGGGTCAT	GAGTCCTTCCACGATACCAA
PVT1	TTGGCACATACAGCCATCAT	CAGTAAAAGGGGAACACCA
VEGFA	CTGTCTTGGGTGCATTGGAG	ACCAGGGTCTCGATTGGATG
ALKBH5	CGGCGAAGGCTACACTTACG	CCACCAGCTTTTGGATCACCA
CCND1	CCCTCGGTGTCCTACTTCAAATGT	GGAAGCGGTCCAGGTAGTTCAT
CDK1	TTTTCAGAGCTTTGGGCACT	CCATTTTGCCAGAAATTCGT
MMP2	CTGCGGTTTTCTCGAATCCATG	GTCCTTACCGTCAAAGGGGTATCC
MMP9	GAGGCGCTCATGTACCCTATGTAC	GTTCAGGGCGAGGACCATAGAG
Vimantin	AAGTTTGCTGACCTCTCTGAGGCT	CTTCCATTTCACGCATCTGGCGTT

### RNA interference

Small interfering RNAs (siRNAs) targeting ALKBH5 (si1 5′-CTGCAAGTTCCAGTTCAA-3′ and si2 5′-GGGCCAAGCGCAAGTATCA-3′), negative control (NC) siRNA (5′-TTCTCCGAACGTGTCACGT-3′), and siRNA targeting PVT1 (5′-CAGCCATCATGATGGTACT-3′) were purchased from General Biosystems (Anhui, China). The control plasmid pcDNA3.1 and overexpression plasmid pcDNA3.1-PVT1 were obtained from General Biosystems. The cells were cultured in six-well plates, and the siRNAs or plasmids were transfected using Lipofectamine 2000 reagents (Invitrogen, USA) according to the manufacturer’s protocol. At 24 h after transfection, the efficiency of silencing or overexpression was tested by qRT‒PCR.

### CCK-8 assay

CCK-8 (Cell Counting Kit-8, DOJINDO, Japan) assays were performed to evaluate cell proliferation. At 24 h after transfection, the transfected cells were harvested and seeded at a density of 2000 cells/well in 96-well plates. Ten microliters of CCK-8 reagent was added to each well, which contained 100 μL of medium, and cultured for 2 h. Then, the reaction was measured for the optical density at 450 nm by a microplate reader (BioTek Elx800, USA) according to the manufacturer’s instructions. The optical density was measured every 24 h from 0 to 72 h.

### Colony formation assay

The transfected cells were seeded into six-well plates at 600 cells/well and cultured with media containing 10% FBS, 100 U/ml penicillin and 100 µg/ml streptomycin. The media were replaced every three days. After 14 days, the colonies were washed twice with PBS, fixed with methanol for 20 min and stained with 0.1% crystal violet for 20 min. Then, the stained colonies were photographed and counted.

### Transwell assay

The transwell assay was performed using 24-well plates with 8 μm pore size chamber inserts (Millipore, USA) to evaluate cell migration. Transfected cells (3 × 10^4^ or 5 × 10^4^) diluted with 200 μL serum-free medium were seeded into the upper chambers of transwell plates. Then, the upper chambers were placed into the lower chambers in 24-well plates, which contained 800 μL medium with 10% FBS. After culturing for 24 h, the cells were fixed using methanol for 20 min and stained using 0.1% crystal violet for 20 min. Then, the cells were imaged under a microscope with 10 × objective lens. The number of migrated cells of each image was counted manually for assessing the ability of cell migration.

### Tube formation experiment

Fifty microliters of precooled Matrigel (Corning, USA) was added to 96-well plates and incubated for 30 min at 37 °C for hardening. Human umbilical vein endothelial cells (HUVECs; 2 × 10^4^ cells) and the supernatant of transfected cells were added to each well and incubated at 37 °C for 6–12 h. The tube-like structures were imaged under a microscope and quantified with ImageJ software.

### Western blotting

Total protein was extracted from the transfected cells that were lysed with radioimmunoprecipitation assay (RIPA, Beyotime, China) lysis buffer. Sodium dodecyl sulfate–polyacrylamide gel electrophoresis (SDS‒PAGE) was used to separate protein samples and electrotransferred to polyvinylidene fluoride (PVDF) membranes (Millipore, Schwalbach, Germany), which were blocked with 5% skim milk. Then, the membranes were incubated with a primary antibody (BOSTER, China) against ALKBH5 (ALKBH5, 1:1000) or GAPDH (1:10,000) at 4 ℃ overnight. Subsequently, the membranes were incubated with the secondary antibody (1:5000, BOSTER, China) for 1 h at room temperature. The protein bands were visualized with a BeyoECL plus kit (Beyotime, China) after washing.

### Zebrafish husbandry

The adult zebrafish were maintained at 28 °C and a 14 h–10 h light–dark cycle in a fish auto culture system (Haisheng, China). Zebrafish embryos were harvested and cultured in 10% Hank’s solution composed of 140 mM NaCl, 5.4 mM KCl, 0.25 mM Na_2_HPO_4_, 0.44 mM KH_2_PO_4_, 1.3 mM CaCl_2_, 1.0 mM MgSO_4_ and 4.2 mM NaHCO_3_ (pH 7.2). At 48 h postfertilization (hpf), wild-type AB or Tg(fli1a: EGFP) zebrafish larvae were used for tumor xenograft models in our study [45]. Zebrafish handling procedures were approved by Kangda College of Nanjing Medical University.

### Zebrafish xenograft model

Before injection, the transfected cells were labeled with CM-DiI (Invitrogen, USA) [46]. Cultured cells were collected and washed with PBS for three times, then the cells were stained with CM-DiI (1 μg/μL in PBS) at 37 °C for 5 min, following by 15 min at 4 °C. The stained cells were rinsed three times with PBS and then examined by fluorescence microscopy. The 48-hpf zebrafish larvae were fixed with 1.2% low-melting gel (Promega, USA), and approximately 400 labeled cells were injected into the perivitelline space (PVS) of zebrafish larvae under a microinjector (Picosprizer III, USA). Then, the zebrafish larvae were cultured at 34 ℃ after injection. At 1 day post injection (dpi), similar sizes of fluorescence areas were selected by stereotype microscopy (MVX10, Olympus, Japan) for further research and cultured at 34 °C until the end of the experiment.

At 4 dpi, the zebrafish larvae were fixed with 1.2% low-melting gel, and the yolk and trunk were imaged by a stereotype microscope (MVX10, Olympus, Japan) or confocal microscope using a 20X water-immersion objective (Fluoview 3000, Olympus, Japan). The resolution of the images was 1600 × 1200 (MVX10) or 1024 × 1024 pixels. The fluorescence area of the yolk of zebrafish larvae was quantified to assess cell proliferation, and the fluorescence area of the trunk of zebrafish larvae was quantified to evaluate cell migration after knockdown of ALKBH5 or PVT1 in lung cancer cells.

For angiogenesis studies, approximately 1000 CM-DiI-labeled cells were injected into the perivitelline space (PVS) of zebrafish larvae. At 2 dpi, the zebrafish larvae were fixed with 1.2% low-melting gel, and the additional branches and sprouts of subintestinal vessels (SIVs) of zebrafish larvae were imaged by confocal microscopy using a 20X water-immersion objective (Fluoview 1000, Olympus, Japan) [47, 48].

### RNA stability assay

The cells were cultured in medium containing 5 μg/mL actinomycin D (Sigma, USA). RNA was extracted with TRIzol reagent, and mRNA expression was detected using qRT‒PCR at 0 h, 2 h and 4 h. Then, expression was measured by qRT‒PCR and calculated using the 2^−∆∆Ct^ method.

### RNA immunoprecipitation (RIP)

The Magna RIP^™^ RNA-Binding Protein Immunoprecipitation Kit (Millipore, USA) is used for RNA immunoprecipitation. According to the manufacturer's instructions, RIP lysis buffer was prepared to treat 2X10^7^ A549 cells. The cell lysates were incubated with containing ALKBH5 antibody or normal rabbit IgG-coupled magnetic beads overnight at 4 °C, and the RNA protein/antibody complexes were then immunoprecipitated by magnetic beads. RNA is extracted from the precipitated complex for qRT‒PCR.

### Statistical analysis

All statistical data were analyzed using unpaired Student’s t tests or Tukey’s multiple comparisons tests after one-way ANOVA, and figures were generated using GraphPad Prism 8.0 software. The fluorescent area of zebrafish larvae was quantified using ImageJ software. Values of P < 0.05 were considered to be statistically significant. All results are presented as the mean ± SEM.

## Results

### ALKBH5 is upregulated in lung cancer tissues and associated with poor prognosis

**Fig. 1:**
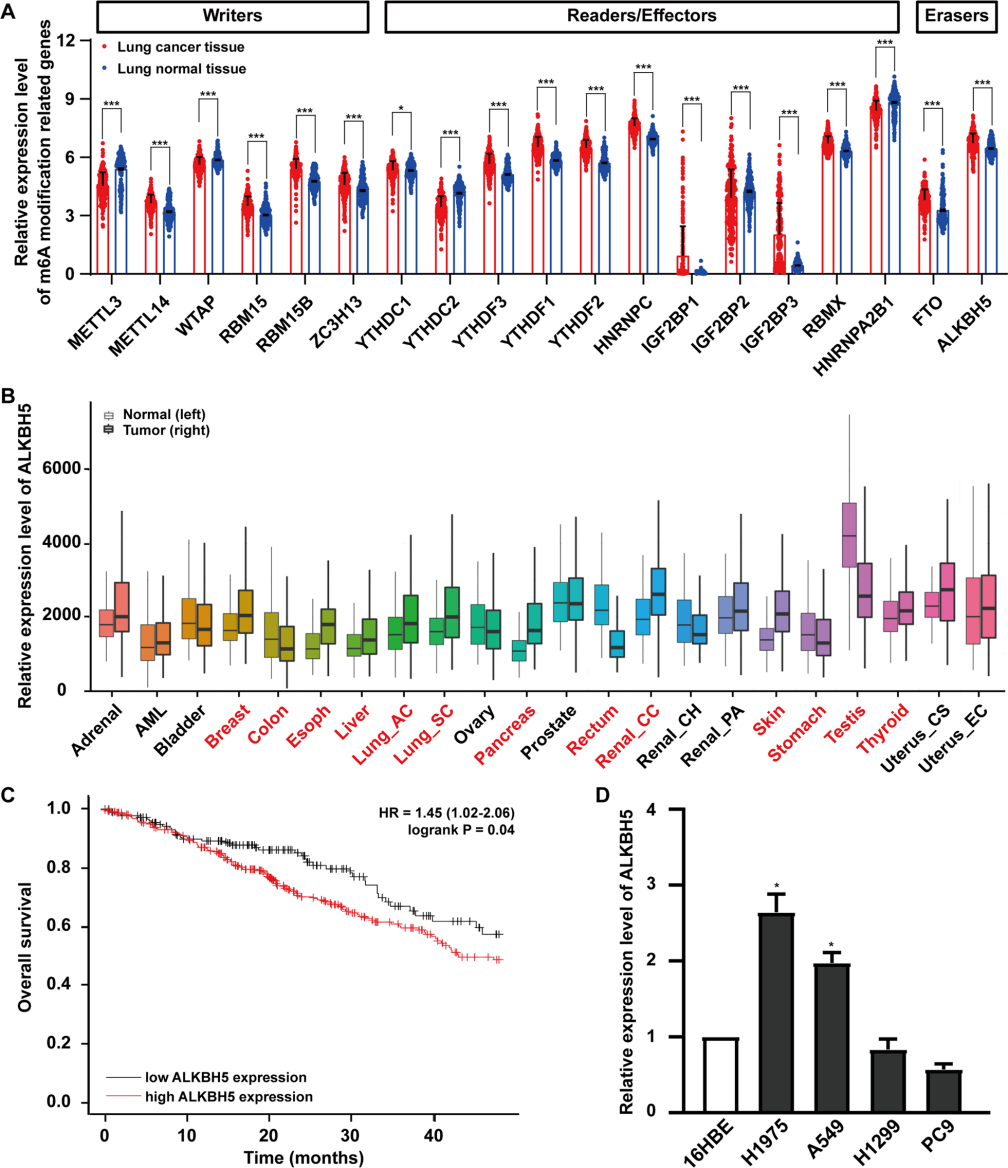
ALKBH5 is upregulated in lung cancer tissues and associated with poor prognosis. **A** The expression levels of m6A modification-related genes in lung cancer tissues and normal tissues obtained by analyzing the TCGA database and GTEx database. **B** ALKBH5 expression in different tumors was analyzed using the TNM plot website. Tumors in red represent significant differences in expression levels between tumors and normal tissues. **C** Kaplan‒Meier analysis was used to show the correlation between ALKBH5 expression levels and overall survival of patients with lung cancer. **D** The expression of ALKBH5 was measured by qRT‒PCR in four lung cancer cell lines (A549, H1299, H1975, PC9) compared with the human bronchial epithelial cell 16HBE. *: P < 0.05, ***: P < 0.001

We analyzed the expression levels of m6A modification-related genes in the TCGA database and GTEx database, including 513 lung cancer tissues and 637 normal tissues. The data showed that most m6A modification-related genes were highly expressed in lung cancers (Fig. 1A). To study the function of m6A-related genes in lung cancer, we chose ALKBH5, which is one of the two m6A mRNA demethylases. We found that ALKBH5 was highly expressed in lung cancer tissues compared with normal tissues (Fig. 1A). Next, we analyzed the expression levels of ALKBH5 in different tumor tissues and normal tissues using the TNM plot website and found that ALKBH5 was highly expressed in most tumor tissues, including lung adenocarcinoma (LUAD) and lung squamous cell carcinoma (LUSC; Fig. 1B). Next, a Kaplan‒Meier analysis revealed that lung cancer patients with high expression levels of ALKBH5 had shorter overall survival (OS) than those with low expression levels of ALKBH5 (Fig. 1C). We then detected the expression of ALKBH5 in four lung cancer cell lines (A549, H1299, H1975, PC9) and human bronchial epithelial cell line (16HBE), and found that ALKBH5 was more highly expressed in H1975 and A549 cells than in 16HBE cells (Fig. 1D). These data show that ALKBH5 is upregulated in lung cancer and associated with poor prognosis of lung cancer patients.

### Knockdown of ALKBH5 inhibits the proliferation and migration of lung cancer cells in vitro

**Fig. 2:**
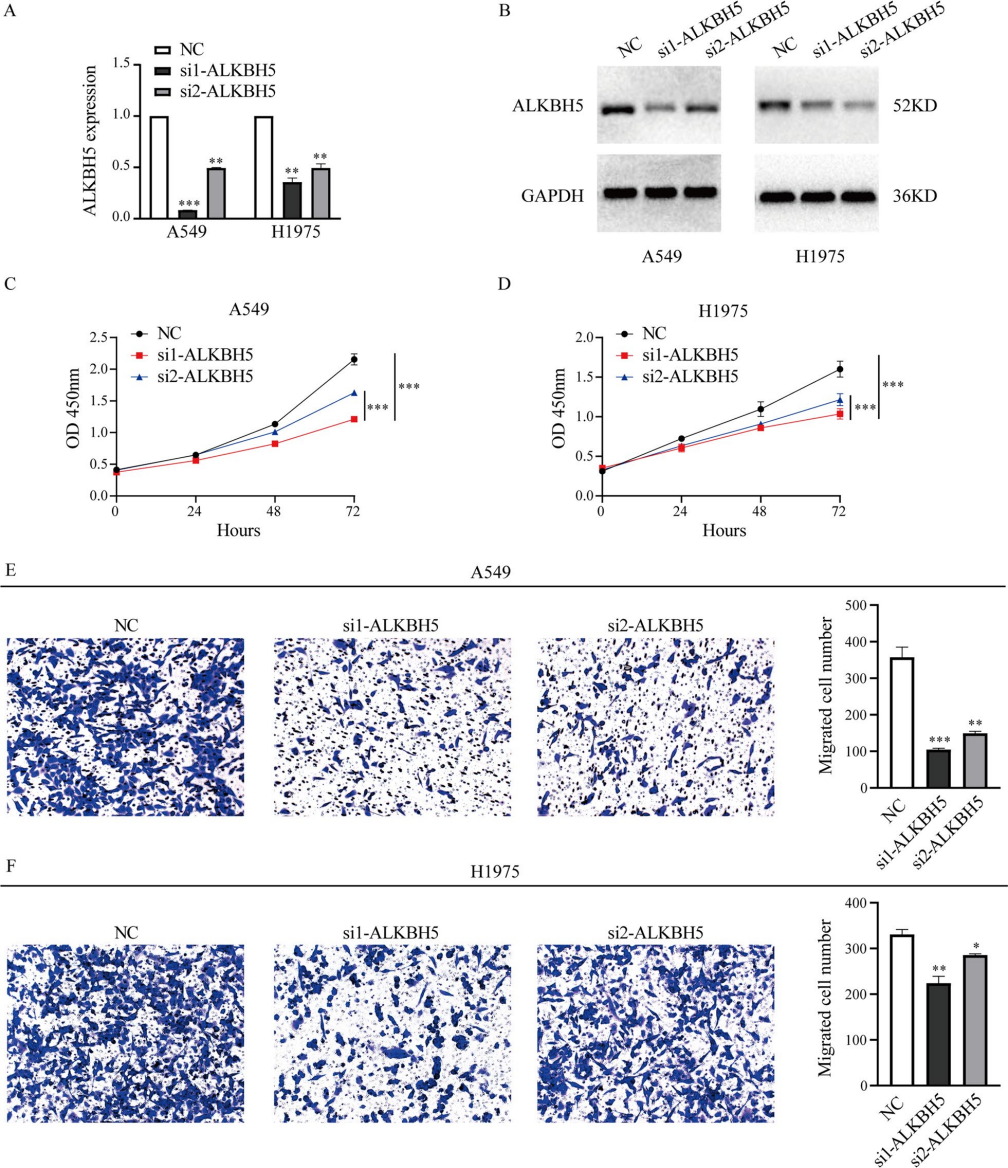
Knockdown of ALKBH5 inhibits the proliferation and migration of lung cancer cells in vitro. **A** The knockdown efficiency in A549 and H1975 cells after transfection with ALKBH5 siRNAs or NC as shown by qRT‒PCR. **B** The protein expression level of ALKBH5 was detected in A549 and H1975 cells by western blot after transfection with ALKBH5 siRNAs or NC. **C**, **D** CCK-8 assays were performed to evaluate the proliferation of A549 and H1975 cells after knocking down ALKBH5. **E**, **F** Transwell assays were performed to evaluate migration in A549 and H1975 cells after knocking down ALKBH5. *: P < 0.05, **: P < 0.01, ***: P < 0.001

To study the biological functions of ALKBH5 in lung cancer cells, we first transfected ALKBH5 siRNAs or NC siRNA into A549 and H1975 cells to silence ALKBH5. The silencing efficiency of si1-ALKBH5 was 83.6% and 50.5% in A549 and H1975 cells, respectively, and the efficiency of si2-ALKBH5 was 50.5% and 39.9% in A549 and H1975 cells, respectively (Fig. 2A). Western blot assays were also performed to verify the silencing effects of ALKBH5 in A549 and H1975 cells, and the expression of ALKBH5 at the protein level was downregulated by ALKBH5 siRNAs (Fig. 2B). Next, CCK-8 assays showed that cell proliferation was dramatically inhibited when ALKBH5 was silenced in A549 and H1975 cells (Fig. 2C, D). Transwell assays showed that cell migration was markedly suppressed when ALKBH5 was downregulated in A549 and H1975 cells (Fig. 2E, F) Compared with lung cancer cell lines, we also examined the roles of ALKBH5 in 16HBE cells, but we found ALKBH5 knockdown did not affect the proliferation and migration of 16HBE cells obviously (Additional file 1: Fig. S1). These results demonstrate that ALKBH5 regulates the proliferation and migration of lung cancer cells in vitro.

### Knockdown of ALKBH5 inhibits the growth and metastasis of lung cancer cells in vivo

**Fig. 3:**
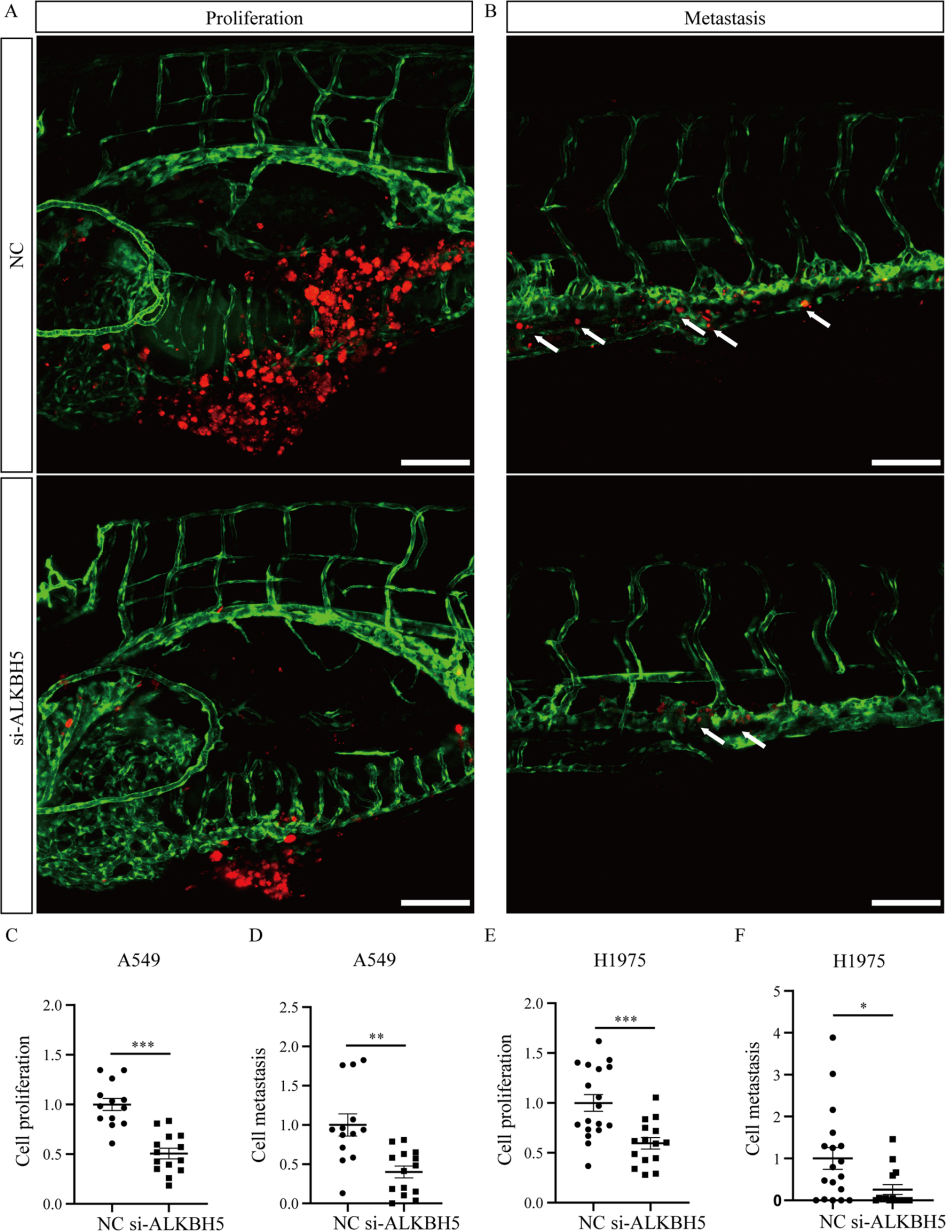
Knockdown of ALKBH5 inhibits the proliferation and metastasis of lung cancer cells in vivo. **A**, **B** The CM-DiI-positive signals in the yolk and trunk of zebrafish were imaged by confocal microscopy at 4 days postinjection of A549 cells transfected with si-ALKBH5 or NC. The red signals indicate the cancer cells labeled CM-DiI. The green signals indicate the blood vessels that expressed EGFP. The white arrows represent the metastatic cells. Scale bar: 100 μm. **C**, **D** Statistical analysis of the proliferation and metastasis of A549 cells after knockdown of ALKBH5. NC: n = 13, si-ALKBH5: n = 14. **E**, **F** Statistical analysis of the proliferation and metastasis of H1975 cells after knockdown of ALKBH5. NC: n = 13, si-ALKBH5: n = 14. *: P < 0.05, **: P < 0.01, ***: P < 0.001

To investigate the functional roles of ALKBH5 in lung cancer cells in vivo, zebrafish xenograft models were used. Before injection, A549 and H1975 cells transfected with si-ALKBH5 or NC were harvested and labeled with CM-DiI. Approximately 400 labeled cells were transplanted into the PVS of 48-hpf Tg (fli1a: EGFP) zebrafish larvae, in which the vascular endothelial cells were labeled with EGFP. At 1 dpi, the samples with similar size fluorescent area were selected from all zebrafish xenografts by fluorescence microscopy for further research. At 4 dpi, the images were acquired via stereomicroscopy and confocal microscopy, including the yolk and trunk of zebrafish larvae. The area of CM-DiI signals in the yolk represented the proliferation, and the area of CM-DiI signals in the trunk represented the metastasis of A549 cells (Fig. 3A, B). The results of zebrafish xenograft models of lung cancers showed that silencing ALKBH5 inhibited the proliferation and metastasis of A549 cells in vivo (Fig. 3C, D). Similarly, knockdown of ALKBH5 also suppressed the proliferation and metastasis of H1975 cells in zebrafish xenografts (Fig. 3E, F). These results demonstrate that silencing ALKBH5 suppresses the proliferation and metastasis of lung cancer cells in vivo.

### Knockdown of ALKBH5 inhibits the angiogenesis of lung cancer cells in vitro and in vivo

**Fig. 4:**
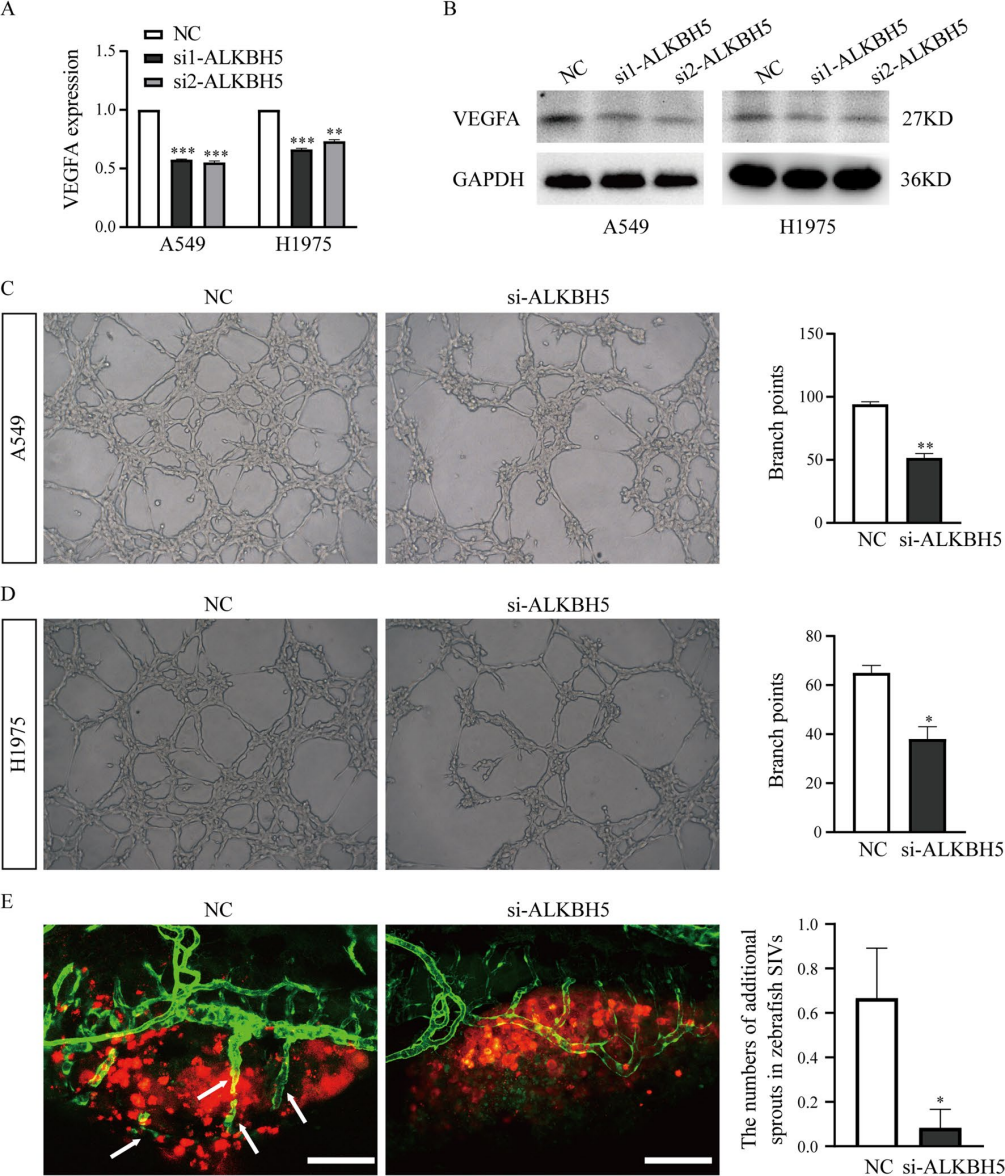
Knockdown of ALKBH5 inhibits the angiogenesis of lung cancer in vitro and in vivo. **A**, **B** The expression of VEGFA in A549 and H1975 cells when ALKBH5 was knocked down was detected with qRT‒PCR and western blotting. **C**, **D** HUVECs were cultured with conditioned medium from A549 or H1975 cells transfected with si-ALKBH5 or NC, and the tube formation was quantified. **E** The blood vessels sprouting from subintestinal vessels in zebrafish larvae were analyzed at 48 h after injection of A549 cells with si-ALKBH5 or NC transfection. The red signals indicate the cancer cells labeled CM-DiI. The green signals indicate the blood vessels that expressed EGFP. The white arrows indicate the new blood vessels that sprouted from subintestinal vessels in zebrafish larvae. NC: n = 12, si-ALKBH5: n = 12. Scale bar: 100 μm. *: P < 0.05, **: P < 0.01, ***: P < 0.001

In previous studies, ALKBH5 was shown to regulate postischemic angiogenesis through destabilization of WNT5A mRNA in an m6A-dependent manner [49], and it can also regulate SPHK1-dependent endothelial cell angiogenesis following acute ischemic stress [50]. These studies suggest that ALKBH5 might affect prognosis by regulating tumor angiogenesis. However, whether ALKBH5 regulates angiogenesis in human cancers is still unclear. Due to the importance of VEGFA in angiogenesis, we first analyzed the correlation of expression between ALKBH5 and VEGFA in lung cancer tissues using the GEPIA website and found that the expression levels of ALKBH5 were positively correlated with VEGFA in lung cancer tissues (Additional file 1: Fig. S2). Next, we detected the expression of VEGFA in A549 and H1975 cells by qRT‒PCR and western blotting and found that VEGFA expression was decreased at both the transcriptional and translational levels when ALKBH5 was knocked down (Fig. 4A, B). Subsequently, a tube formation assay of human umbilical vein endothelial cells (HUVECs) was performed to study angiogenesis in vitro. The conditioned media of A549 or H1975 cells transfected with si-ALKBH5 or NC was collected to treat HUVECs, and we found that tube formation was inhibited when ALKBH5 was silenced in the two lung cancer cell lines (Fig. 4C, D and Additional file 1: Fig. S3).

Next, we also examined the angiogenesis of lung cancers using zebrafish xenografts. A549 cells transfected with si-ALKBH5 or NC were harvested and labeled with CM-DiI, and approximately 1000 cancer cells were injected into the PVS of 48-hpf Tg (fli1a:EGFP) zebrafish larvae. At 1 dpi, samples with similar size fluorescent area were also selected from all zebrafish xenografts for further research. At 2 dpi, images of the yolk of zebrafish xenografts were acquired via confocal microscopy. We found that transplanting A549 cells with NC siRNA transfection induced additional blood vessels that sprouted from the subintestinal vein (SIV) of zebrafish larvae, but this induction was significantly suppressed when transplanting A549 cells with si-ALKBH5 transfection (Fig. 4E). These data demonstrate that knockdown of ALKBH5 inhibits the angiogenesis of lung cancers in vitro and in vivo.

### Knockdown of ALKBH5 decreases the stability of PVT1 but not VEGFA in lung cancer cells

**Fig. 5:**
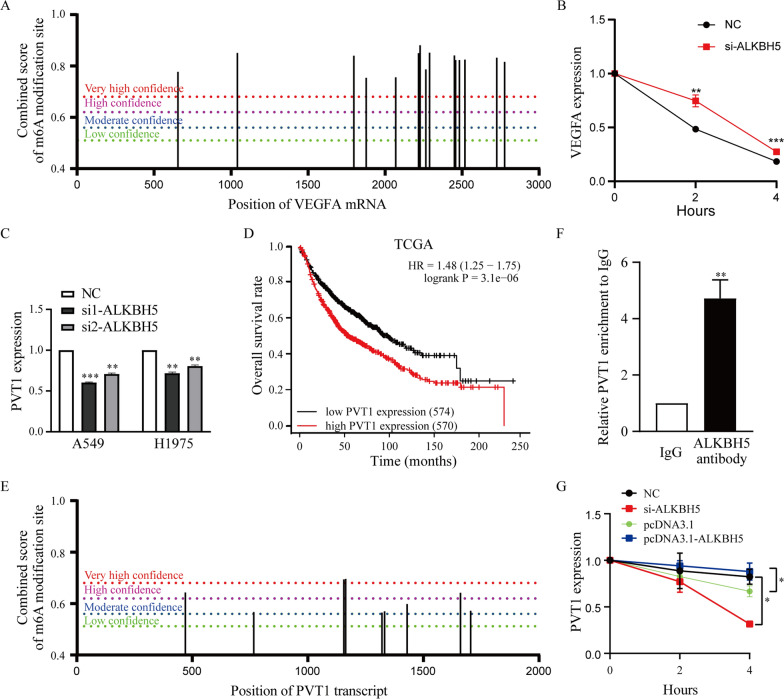
Knockdown of ALKBH5 decreases the stability of PVT1 but not VEGFA mRNA in lung cancer cells. **A** m6A modification sites of VEGFA mRNA were predicted by SRAMP. **B** The stability of VEGFA over time in A549 cells transfected with si-ALKBH5 was measured by qRT‒PCR relative to time point 0. **C** qRT‒PCR was used to detect the expression of PVT1 in A549 and H1975 cells after silencing ALKBH5. **D** Kaplan‒Meier analysis was used to analyze the correlation between PVT1 expression levels and overall survival of patients with lung cancer from the TCGA database. **E** m6A modification sites of PVT1 were predicted by SRAMP. **F** RIP experiments were performed in A549 cells and the coprecipitated RNA was subjected to qRT-PCR for PVT1. Expression levels of PVT1 are as fold enrichment in ALKBH5 antibody relative to IgG immunoprecipitates. **G** The stability of PVT1 over time in A549 cells transfected with si-ALKBH5 or pcDNA3.1-ALKBH5 was measured by qRT‒PCR relative to time point 0. *: P < 0.05, **: P < 0.01, ***: P < 0.001

To study the mechanism of ALKBH5 in tumor angiogenesis in lung cancer cells, we first analyzed the potential m6A modification sites of VEGFA mRNA. We found that VEGFA mRNA had multiple m6A modification sites with high confidence (Fig. 5A). We next used α-amanitin to block RNA synthesis in A549 cells after transfection with si-ALKBH5 or NC and found that knockdown of ALKBH5 did not decrease but rather increased the half-life of VEGFA mRNA slightly in A549 cells (Fig. 5B). These results revealed that ALKBH5 negatively regulated the mRNA stability of VEGFA in lung cancer cells. In contrast to the results that ALKBH5 positively regulated VEGFA expression in lung cancer cells (Fig. 4A, B), the mRNA stability data suggested that there may be a regulatory mediator between ALKBH5 and VEGFA.

A recent study reported that ALKBH5 mediates the m6A modification of PVT1 and increases the stability of PVT1 to promote osteosarcoma tumorigenesis [51]. Thus, we examined the relationship between ALKBH5 and PVT1 in lung cancer and found that knockdown of ALKBH5 decreased the expression levels of PVT1 in A549 and H1975 cells (Fig. 5C). Next, we analyzed the GEO database (GSE30219) using lnCAR software and found that a high PVT1 expression level was also associated with poor overall survival (OS) in lung cancer patients (Additional file 1: Fig. S4). Kaplan‒Meier analysis revealed that lung cancer patients with high expression levels of PVT1 had shorter overall survival (OS) than those with low expression levels of PVT1 in the TCGA database (Fig. 5D). Furthermore, we also analyzed the potential m6A modification sites of PVT1 and found that PVT1 had several m6A modification sites with high confidence (Fig. 5E). To determine whether ALKBH5 mediates the m6A modification of PVT1 in lung cancer cells, we carried out RNA immunoprecipitation (RIP) assays which revealed that ALKBH5 bounded directly to PVT1 in A549 cells (Fig. 5F). We then used α-amanitin to block RNA synthesis in A549 cells after transfection with si-ALKBH5 or pcDNA3.1-ALKBH5 (Additional file 1: Fig. S5) and found that knockdown of ALKBH5 significantly decreased the half-life of PVT1 and overexpression of ALKBH5 slightly increased the half-life of PVT1 in A549 cells (Fig. 5G). Taken together, these results suggest that silencing ALKBH5 decreased the expression level of PVT1 by regulating its stability in lung cancer cells.

### PVT1 promotes the proliferation and metastasis of lung cancer cells in vitro and in vivo

**Fig. 6:**
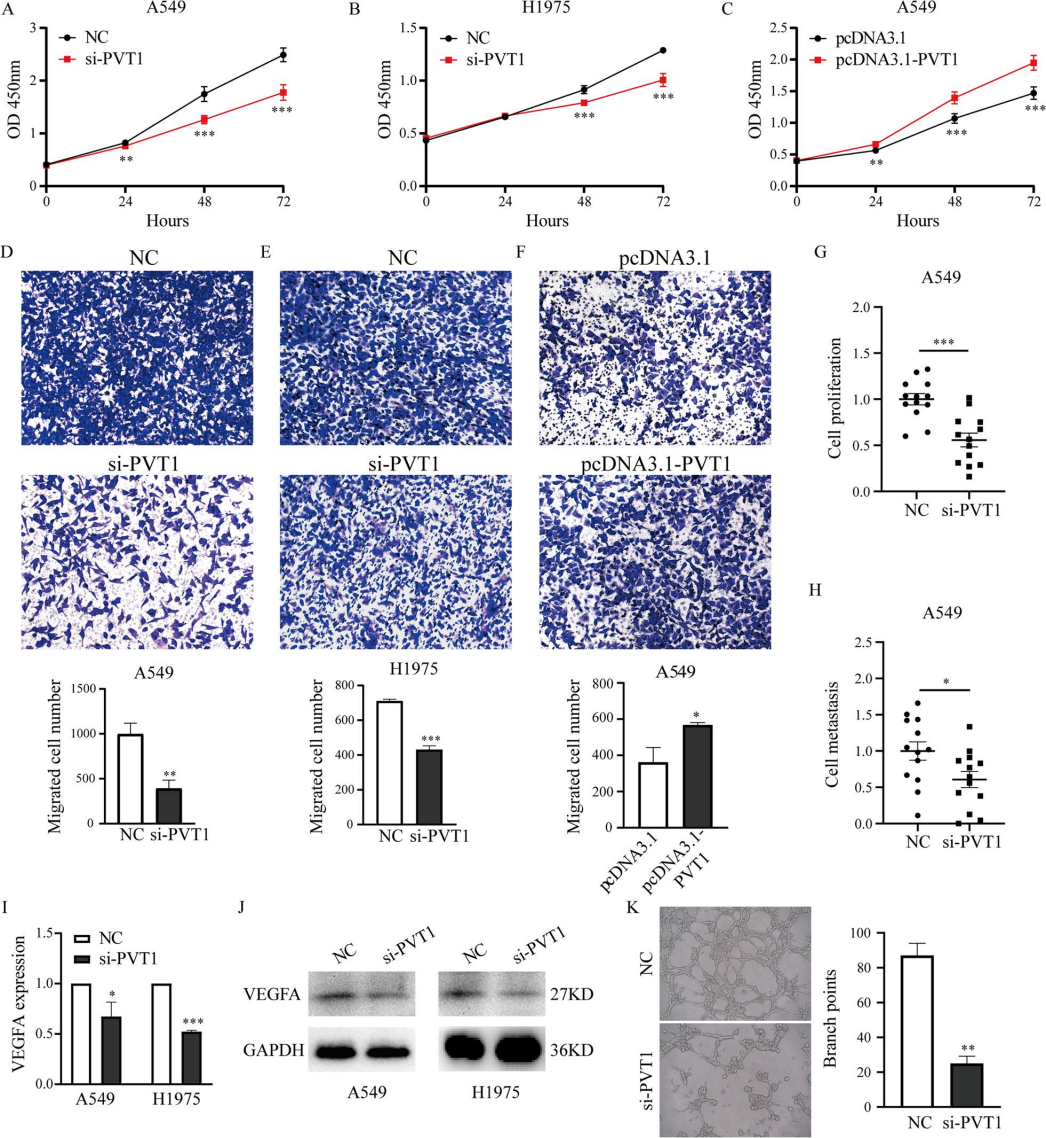
PVT1 promotes the proliferation, metastasis and angiogenesis of lung cancer cells. **A**–**C** CCK-8 assays were performed to evaluate the proliferation of lung cancer cells when PVT1 was knocked down or overexpressed. **D**-**F** Cell migration was evaluated by transwell assays when PVT1 was knocked down or overexpressed in lung cancer cells. **G, H** Statistical analysis of proliferation and metastasis in A549 cells after silencing PVT1 in zebrafish xenografts. NC: n = 13, si-PVT1: n = 13. **I, J** VEGFA expression in A549 and H1975 cells with PVT1 knockdown was measured by qRT‒PCR and western blotting. **K** HUVECs were cultured with conditioned medium from A549 cells transfected with si-PVT1 or NC, and the tube formation was quantified. *: P < 0.05, **: P < 0.01, ***: P < 0.001

To verify the function of PVT1 in lung cancer cells, we also detected the expression levels of PVT1 in four lung cancer cell lines (A549, H1299, H1975, PC9) and 16HBE and found that PVT1 was also highly expressed in H1975 and A549 cells (Additional file 1: Fig. S6). Then, we transfected si-PVT1 or NC into A549 and H1975 cells to knockdown PVT1, and the silencing efficiencies were 65.8% and 51.7% in H1975 and A549 cells, respectively (Additional file 1: Fig. S7A). In addition, the plasmid pcDNA3.1-PVT1 was transfected into A549 cells to overexpress PVT1, and the overexpression efficiency was 21.9 times that of the control plasmid (transfection with the pcDNA3.1 vector; Additional file 1: Fig. S7B). CCK-8 assays and colony formation assays showed that cell proliferation was dramatically inhibited when PVT1 was knocked down (Fig. 6A, B and Additional file 1: Fig. S8A, B). In contrast, PVT1 overexpression increased the proliferation of A549 cells (Fig. 6C and Additional file 1: Fig. S8C). Transwell assays showed that inhibition of PVT1 in A549 and H1975 cells markedly suppressed cell migration (Fig. 6D, E). In contrast, migration was enhanced when PVT1 was overexpressed in A549 cells (Fig. 6F). Subsequently, we also investigated the functions of PVT1 using zebrafish xenograft models and found that the growth and metastasis of lung cancer cells were suppressed when PVT1 was knocked down (Fig. 6G, H). These results demonstrate that PVT1 promotes the proliferation and metastasis of lung cancer cells in vitro and in vivo.

### Knockdown of PVT1 decreases the expression of VEGFA and inhibits angiogenesis in lung cancer

We next investigated the roles of PVT1 in lung cancer angiogenesis. We first analyzed the correlation of expression between PVT1 and VEGFA using the GEPIA website and found that they were significantly correlated with each other in lung cancer tissues (Additional file 1: Fig. S9). Next, we analyzed the expression of VEGFA in A549 and H1975 cells transfected with si-PVT1 or NC and found that knockdown of PVT1 decreased VEGFA expression levels at the transcriptional and translational levels (Fig. 6I, J). Subsequently, the tube formation assay showed that tube formation was inhibited when PVT1 was silenced in A549 cells (Fig. 6K and Additional file 1: Fig. S10). These results demonstrate that knockdown of PVT1 decreases the expression of VEGFA and inhibits the angiogenesis of lung cancer.

### PVT1 overexpression partially restores the proliferation, migration and angiogenesis that were suppressed by ALKBH5 knockdown in lung cancer cells

**Fig. 7:**
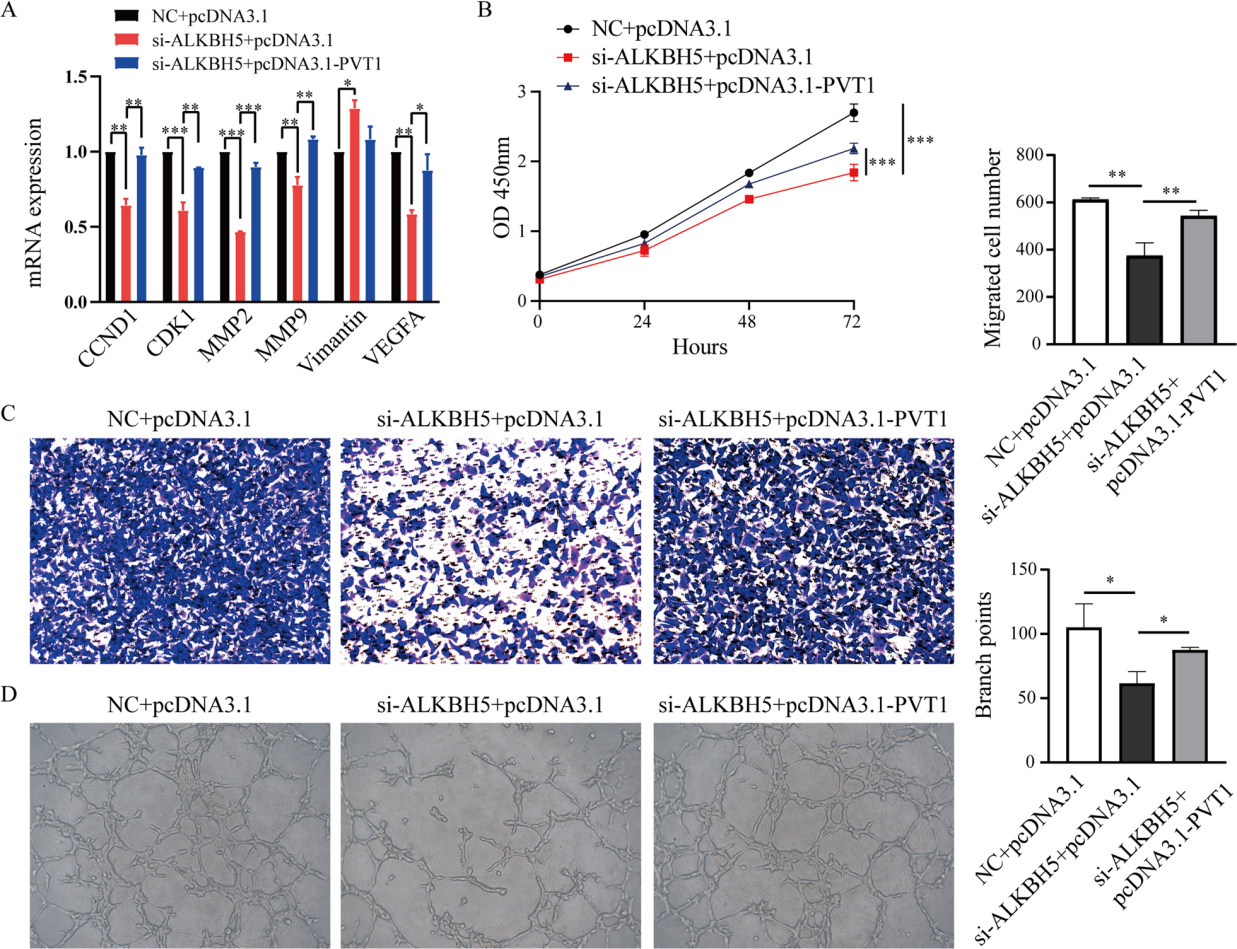
PVT1 overexpression partially restores the proliferation, migration and angiogenesis of lung cancer following suppression by ALKBH5 knockdown. **A** PVT1-regulated genes were examined by qRT-PCR in A549 cells cotransfected with si-ALKBH5 or NC and the pcDNA3.1 or pcDNA3.1-PVT1 plasmid. **B** CCK-8 assays were performed to assess proliferation in A549 cells cotransfected with si-ALKBH5 or NC and the pcDNA3.1 or pcDNA3.1-PVT1 plasmid. **C** Transwell assays were performed to assess the migration of A549 cells cotransfected with si-ALKBH5 or NC and the pcDNA3.1 or pcDNA3.1-PVT1 plasmid. **D** HUVECs were treated with conditioned medium from A549 cells cotransfected with si-ALKBH5 or NC and the pcDNA3.1 or pcDNA3.1-PVT1 plasmid, and the tube formation was quantified. *: P < 0.05, **: P < 0.01, ***: P < 0.001

Finally, we investigated the functional mechanism between ALKBH5 and PVT1 in lung cancer cells. We cotransfected si-ALKBH5 and pcDNA3.1-PVT1 into A549 cells, and then examined the expression of a serious of PVT1-regulated genes (CCND1, CDK1, MMP2, MMP9, Vimantin and VEGFA) [52–56]. We found that most of these genes were downregulated when ALKBH5 was knocked down, but PVT1 overexpression could partially rescue the expression of these genes (Fig. 7A). Next, CCK-8 assay showed the suppression effects of cell proliferation caused by ALKBH5 knockdown were partially restored by PVT1 overexpression in A549 cells (Fig. 7B). Similarly, transwell assay showed that PVT1 overexpression impaired the inhibitory effect on cell migration when ALKBH5 was silenced (Fig. 7C). Tube formation assay also showed that the tube formation ability, which was suppressed by silencing ALKBH5, was partially rescued by PVT1 overexpression in lung cancer cells (Fig. 7D and Additional file 1: Fig. S11). These results indicate that ALKBH5 may regulate the proliferation, migration and angiogenesis of lung cancer through PVT1.

## Discussion

In this study, we identified the m6A demethylase ALKBH5 using a public database of lung cancer tissues, and found that it was highly associated with poor overall survival of lung cancer patients. Angiogenesis is an important event for tumor growth and metastasis. Our study revealed for the first time that ALKBH5 is required for the angiogenesis of lung cancer. Furthermore, we identified that the mechanism by which ALKBH5 regulates proliferation, migration and angiogenesis includes the regulation of the stability of PVT1 in lung cancer cells. These results suggest that ALKBH5 and PVT1 could be indicators of prognosis and potential therapeutic targets for lung cancer patients. However, these findings still require the verification in clinical samples of lung cancer.

The m6A modification has been identified as the most common, highly abundant internal modification of RNA in higher eukaryotes and has been shown to participate in the progression of various tumors [8, 13, 57]. As a demethylase, ALKBH5 has been reported to have oncogenic roles in the progression of lung cancer, osteosarcoma, gastric cancer, colon cancer, and ovarian cancer [25, 26, 51, 58–61]. Here, we investigated the functional roles of ALKBH5 in lung cancer and found that knockdown of ALKBH5 inhibited proliferation and metastasis in lung cancer cells in vitro and in vivo, which is consistent with the results of previous studies [25, 26], indicating that ALKBH5 plays oncogenic roles in lung cancer.

It has also been reported that lncRNAs, acting as regulators, play important roles in the development and progression of various tumors [6, 62]. However, the function of lncRNA m6A modification in cancers needs to be explored further. There are very few studies on ALKBH5-mediated modification of lncRNAs in cancers. Zhang et al. reported that ALKBH5 decreased the methylation of lncRNA NEAT1 to promote gastric cancer progression [58]. Another study showed that ALKBH5-mediated m6A demethylation of the lncRNA RMRP plays an oncogenic role in lung adenocarcinoma [25]. It has also been shown that ALKBH5-mediated m6A modification of lncRNA KCNQ1OT1 regulates the development of LSCC [63], and that ALKBH5-mediated m6A modification of PVT1 promotes the progression of OS [51]. The two studies also showed YTHDF2 could recognize m6A-modified sites of lncRNA KCNQ1OT1 or PVT1 and regulate their stability [51, 63], but whether ALKBH5 regulates the stability of PVT1 via YTHDF2 in lung cancer cells remains to be examined further. Further, we also showed that a serious tumor-related genes could be regulated by ALKBH5-PVT1 axis, but the functional evidence is still lacking. Here, our data indicate that ALKBH5 increased the expression of PVT1 by enhancing its stability and that ALKBH5 can regulate the proliferation, migration and angiogenesis of lung cancer partially through PVT1 in lung cancer cells. These results indicate that the ALKBH5-PVT1 axis plays important roles in lung cancer progression.

In our study, zebrafish xenografts were used as the in vivo model for research. In a previous study, knockdown of ALKBH5 and PVT1 in lung cancer cells inhibited tumor growth in mouse models [64, 65]. Our results showed that silencing ALKBH5 and PVT1 suppressed the proliferation of lung cancer cells in zebrafish xenografts, which is consistent with the results of mouse models, indicating that zebrafish xenografts could be a reliable model for human cancer research. However, a mouse model for studying the metastatic roles of ALKBH5 and PVT1 in lung cancer is lacking. Compared with mouse xenograft models, zebrafish xenografts have many advantages. First, the progression of lung cancer cells in a zebrafish xenograft model can be assessed 96 h postinjection, while 3–5 weeks is required in mouse xenograft models in which shRNA plasmids for gene silencing must be constructed instead of siRNA [66]. Second, cell proliferation and metastasis can be evaluated simultaneously in the same transplanted samples in zebrafish xenografts but not in mouse models. Third, based on the transparent nature of zebrafish larvae with immature immune systems, the behavior of tumor cells at early stages can be monitored in vivo, which is difficult in mouse models.

In addition, a zebrafish xenograft model can also be used to investigate angiogenesis in vivo. In a mouse model, Matrigel plug assays are generally used for the assessment of angiogenesis in vivo, which requires seven days [67]. For zebrafish xenografts, we used EGFP-labeled vascular endothelial cell transgenic lines for transplantation, and it is easy to observe the changes in blood vessels in vivo by fluorescence microscopy [48]. In our study, 48 h after injection of lung cancer cells, the changes in additional blood vessel sprouts of zebrafish SIVs could be observed and quantified. These findings suggest that the zebrafish xenograft model is an effective in vivo model for tumor proliferation, metastasis and angiogenesis, and can be gradually applied in human cancer research.

## Conclusion

In summary, we demonstrated the oncogenic roles of ALKBH5 in lung cancer cells in vitro and in vivo. Furthermore, we found that ALKBH5 promotes angiogenesis in lung cancer. Mechanistically, our results showed that ALKBH5 regulates the expression and RNA stability of PVT1 in lung cancer. Overexpression of PVT1 partially restored the proliferation, migration and angiogenesis suppressed by ALKBH5 knockdown in lung cancer. Our study reveals that ALKBH5 promotes lung cancer progression and angiogenesis through PVT1, suggesting novel therapeutic targets for lung cancer patients.

## Supplementary Information

**Additional file 1: Figure S1.** Knockdown of ALKBH5 does not affect the proliferation and migration of 16HBE cells *in vitro*. **A** CCK-8 assays were performed to evaluate the proliferation of 16HBE cells after knocking down ALKBH5. **B** Transwell assays were performed to evaluate migration in 16HBE cells after knocking down ALKBH5. **Figure S2.** The correlation of expression between ALKBH5 and VEGFA in lung cancer was analyzed using the GEPIA website. **Figure S3. **Three replicates of tube formation with conditioned medium from A549 or H1975 cells transfected with si-ALKBH5 or NC. Red boxes represent the typical cases which are used in Fig.4. **Figure S4.** The overall survival of lung cancer patients with high PVT1 expression and low PVT1 expression in the GSE30219 dataset was analyzed using the lnCAR website. **Figure S5.** The overexpression efficiency of ALKBH5 in A549 cells after transfection with the pcDNA3.1-ALKBH5 or pcDNA3.1 plasmid was measured by qRT‒PCR. **: P < 0.01. **Figure** **S6.** The expression of PVT1 was measured by qRT‒PCR in four lung cancer cell lines (A549, H1299, H1975, PC9) and 16HBE human bronchial epithelial cells. *: P < 0.05, **: P < 0.01. **Figure S7.** **A** The knockdown efficiency of PVT1 in A549 and H1975 cells after transfection with si-PVT1 or NC was measured by qRT‒PCR. **B** The overexpression efficiency of PVT1 in A549 cells after transfection with the pcDNA3.1-PVT1 or pcDNA3.1 plasmid was measured by qRT‒PCR. **: P < 0.01, ***: P < 0.001. **Figure S8.** Colony formation assays were performed to evaluate the proliferation of lung cancer cells when PVT1 was knocked down in A549 (**A**) or H1975 (**B**) cells or overexpressed in A549 cells (C). **: P < 0.01. **Figure S9.** The correlation of expression between PVT1 and VEGFA was analyzed in lung cancer using the GEPIA website. **Figure S10. **Three replicates of tube formation with conditioned medium from A549 cells transfected with si-PVT1 or NC. Red boxes represent the typical cases which are used in Fig.6. **Figure S11. **Three replicates of tube formation with conditioned medium from A549 cells transfected with si-ALKBH5 or NC and pcDNA3.1-PVT1 or pcDNA3.1. Red boxes represent the typical cases which are used in Fig. 7.

## Data Availability

Supporting and raw data are available upon a reasonable request to the corresponding author.

## Abbreviations

m6A: N6-methyladenosine
METTL3: Methyltransferase-like 3
METTL14: Methyltransferase-like 14
METTL16: Methyltransferase-like 16
FTO: Fat mass and obesity-associated protein
ALKBH5: α-Ketoglutarate-dependent dioxygenase homolog 5
LncRNAs: Long noncoding RNAs
PVT1: Plasmacytoma variant translocation
qRT‒PCR: Quantitative real-time PCR
siRNAs: Small interfering RNAs
CCK-8: Cell Counting Kit-8
hpf: Hours postfertilization
dpi: Day postinjection
OS: Overall survival
GAPDH: Glyceraldehyde 3-phosphate dehydrogenase
FBS: Fetal bovine serum
PVS: Perivitelline space
SIVs: Subintestinal vessels
SDS‒PAGE: Sodium dodecyl sulfate–polyacrylamide gel electrophoresis
PVDF: Polyvinylidene fluoride
