# Supplementary material for: The RNA demethylase ALKBH5 promotes the progression and angiogenesis of lung cancer by regulating the stability of the LncRNA PVT1
Source: Cancer Cell Int. 2022 Nov 15;22:353. doi: 10.1186/s12935-022-02770-0 (PMC9664734; doi:10.1186/s12935-022-02770-0)
Supplement: Supplementary file 1 — Additional file 1: Figure S1. Knockdown of ALKBH5 does not affect the proliferation and migration of 16HBE cells in vitro. A CCK-8 assays were performed to evaluate the proliferation of 16HBE cells after knocking down ALKBH5. B Transwell assays were performed to evaluate migration in 16HBE cells after knocking down ALKBH5. Figure S2. The correlation of expression between ALKBH5 and VEGFA in lung cancer was analyzed using the GEPIA website. Figure S3. Three replicates of tube formation with conditioned medium from A549 or H1975 cells transfected with si-ALKBH5 or NC. Red boxes represent the typical cases which are used in Fig.4. Figure S4. The overall survival of lung cancer patients with high PVT1 expression and low PVT1 expression in the GSE30219 dataset was analyzed using the lnCAR website. Figure S5. The overexpression efficiency of ALKBH5 in A549 cells after transfection with the pcDNA3.1-ALKBH5 or pcDNA3.1 plasmid was measured by qRT‒PCR. **: P < 0.01. Figure S6. The expression of PVT1 was measured by qRT‒PCR in four lung cancer cell lines (A549, H1299, H1975, PC9) and 16HBE human bronchial epithelial cells. *: P < 0.05, **: P < 0.01. Figure S7. A The knockdown efficiency of PVT1 in A549 and H1975 cells after transfection with si-PVT1 or NC was measured by qRT‒PCR. B The overexpression efficiency of PVT1 in A549 cells after transfection with the pcDNA3.1-PVT1 or pcDNA3.1 plasmid was measured by qRT‒PCR. **: P < 0.01, ***: P < 0.001. Figure S8. Colony formation assays were performed to evaluate the proliferation of lung cancer cells when PVT1 was knocked down in A549 (A) or H1975 (B) cells or overexpressed in A549 cells (C). **: P < 0.01. Figure S9. The correlation of expression between PVT1 and VEGFA was analyzed in lung cancer using the GEPIA website. Figure S10. Three replicates of tube formation with conditioned medium from A549 cells transfected with si-PVT1 or NC. Red boxes represent the typical cases which are used in Fig.6. Figure S11. Three replicates o [file 12935_2022_2770_MOESM1_ESM.docx]

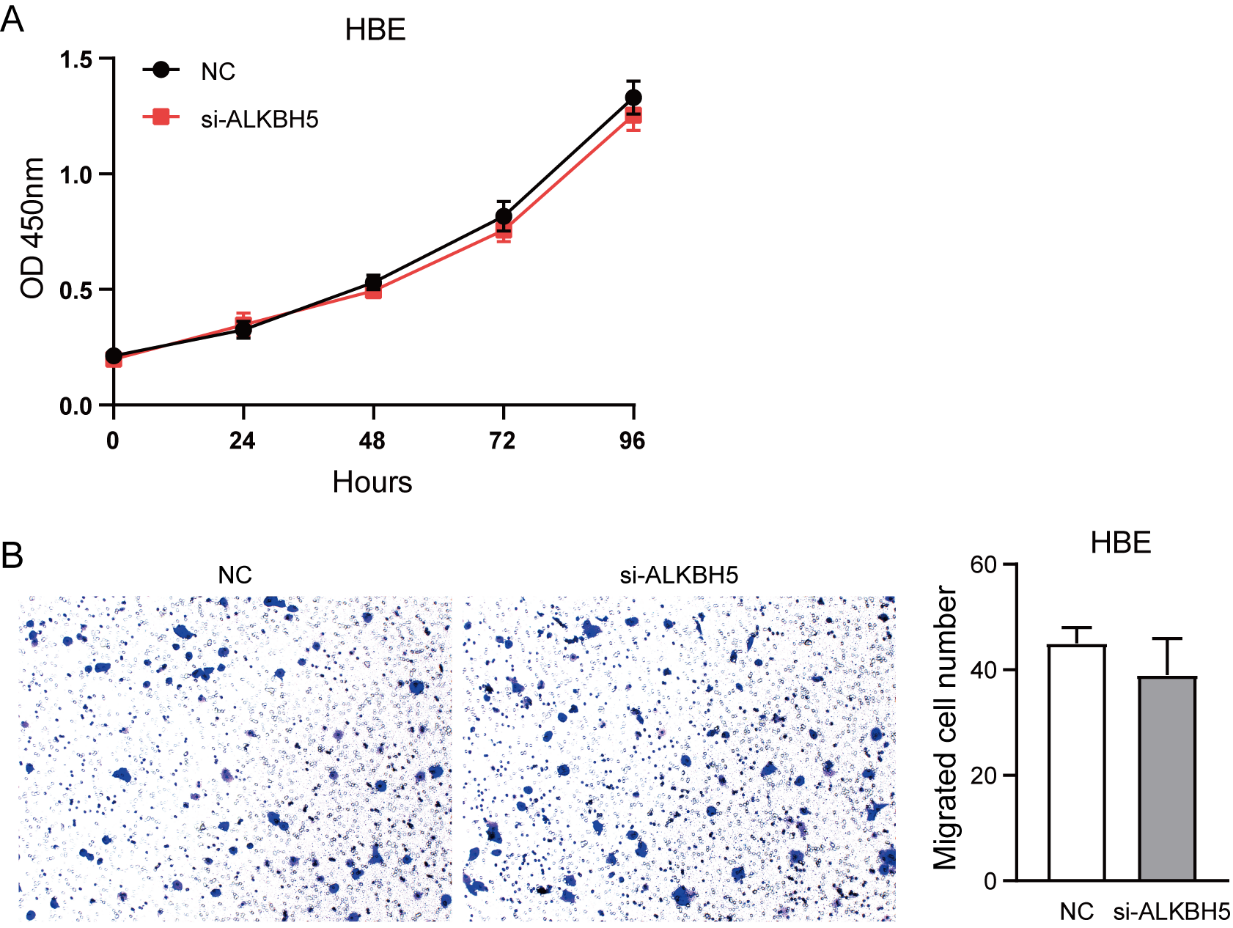


**Fig. S1** Knockdown of ALKBH5 does not affect the proliferation and migration of 16HBE cells *in vitro*. **A** CCK-8 assays were performed to evaluate the proliferation of 16HBE cells after knocking down ALKBH5. **B** Transwell assays were performed to evaluate migration in 16HBE cells after knocking down ALKBH5.


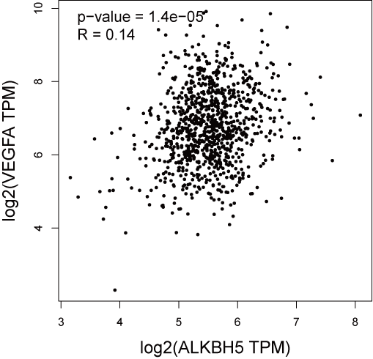


**Fig. S2** The correlation of expression between ALKBH5 and VEGFA in lung cancer was analyzed using the GEPIA website.


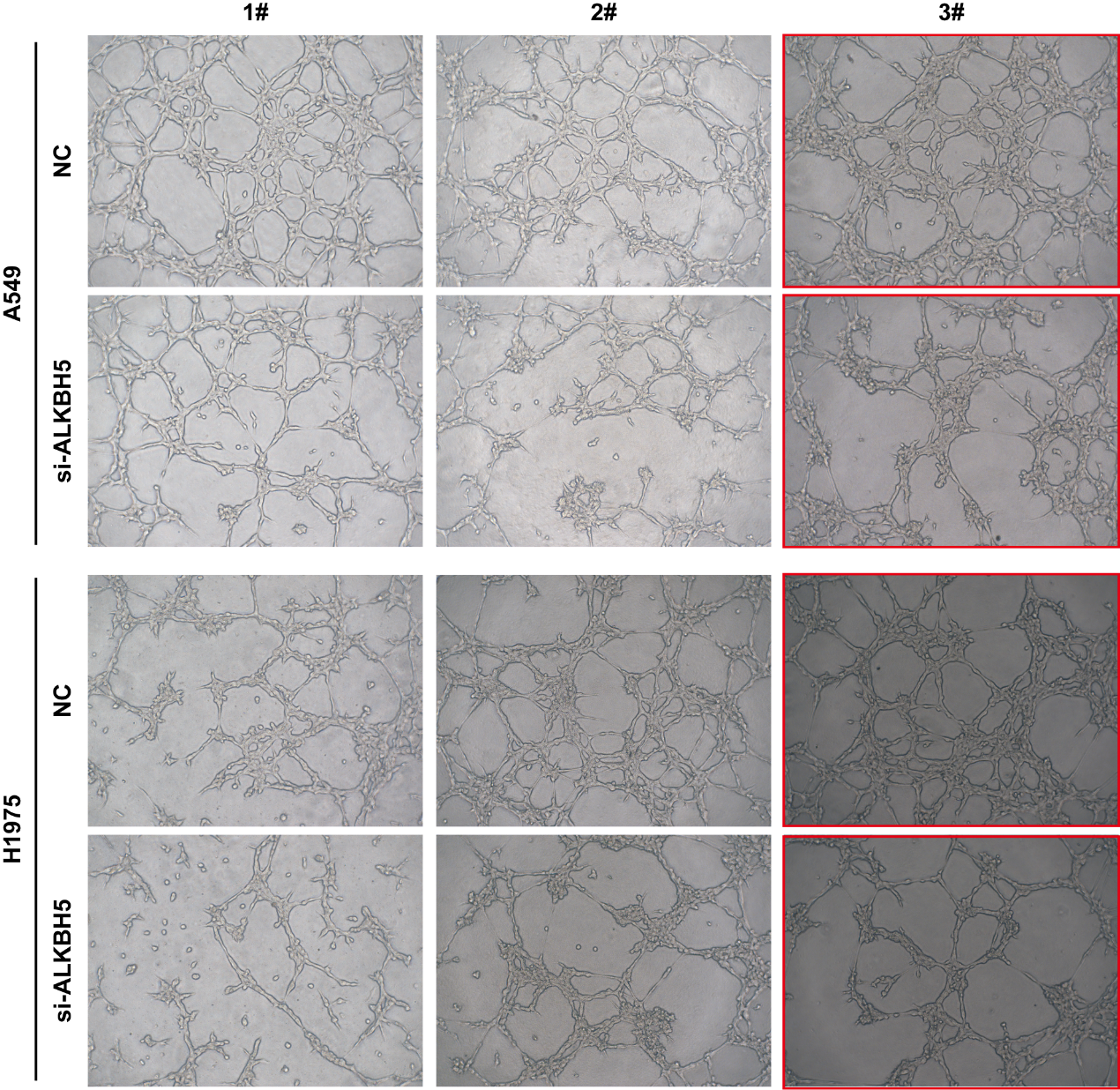


**Fig. S3** Three replicates of tube formation with conditioned medium from A549 or H1975 cells transfected with si-ALKBH5 or NC. Red boxes represent the typical cases which are used in Fig.4.


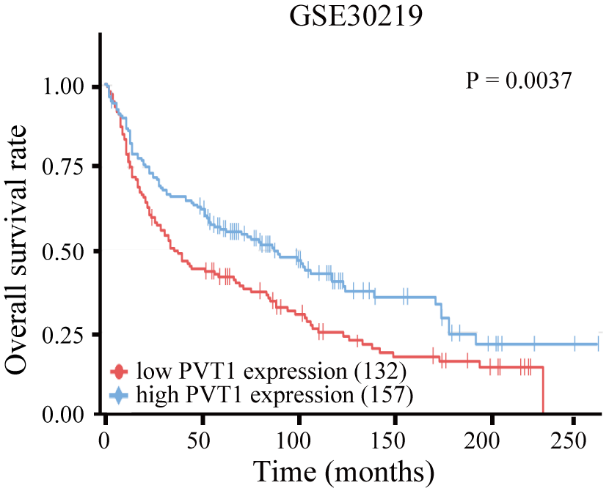


**Fig. S4** The overall survival of lung cancer patients with high PVT1 expression and low PVT1 expression in the GSE30219 dataset was analyzed using the lnCAR website.


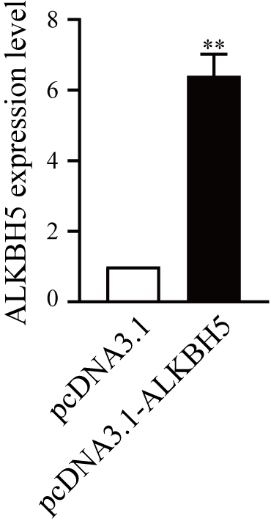


**Fig. S5** The overexpression efficiency of ALKBH5 in A549 cells after transfection with the pcDNA3.1-ALKBH5 or pcDNA3.1 plasmid was measured by qRT‒PCR. **: P < 0.01.


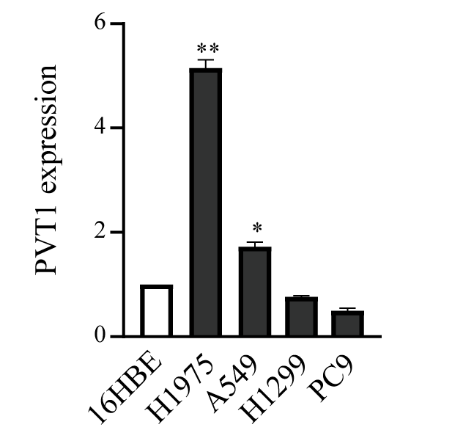


**Fig. S6** The expression of PVT1 was measured by qRT‒PCR in four lung cancer cell lines (A549, H1299, H1975, PC9) and 16HBE human bronchial epithelial cells. *: P < 0.05, **: P < 0.01.


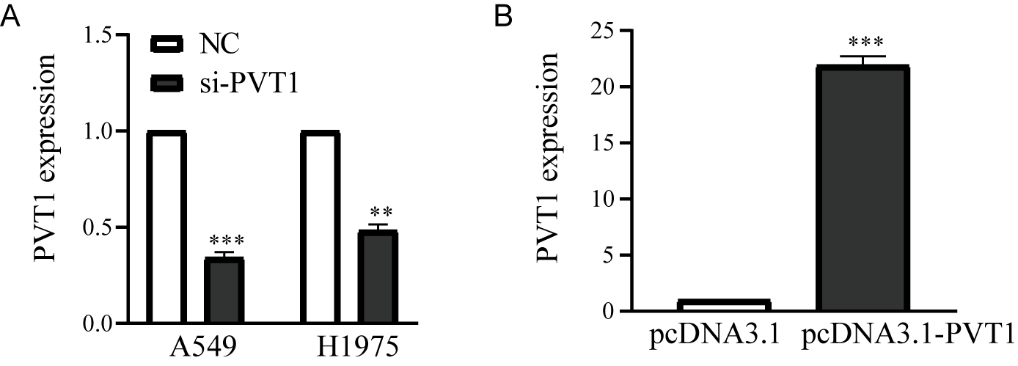


**Fig. S7 A** The knockdown efficiency of PVT1 in A549 and H1975 cells after transfection with si-PVT1 or NC was measured by qRT‒PCR. **B** The overexpression efficiency of PVT1 in A549 cells after transfection with the pcDNA3.1-PVT1 or pcDNA3.1 plasmid was measured by qRT‒PCR. **: P < 0.01, ***: P < 0.001.


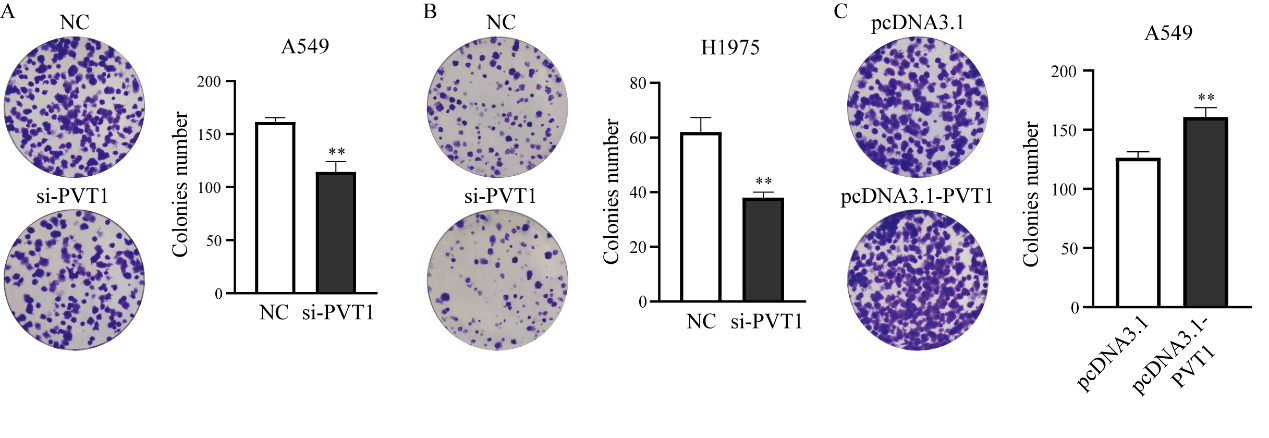


**Fig. S8** Colony formation assays were performed to evaluate the proliferation of lung cancer cells when PVT1 was knocked down in A549 (**A**) or H1975 (**B**) cells or overexpressed in A549 cells (C). **: P < 0.01.


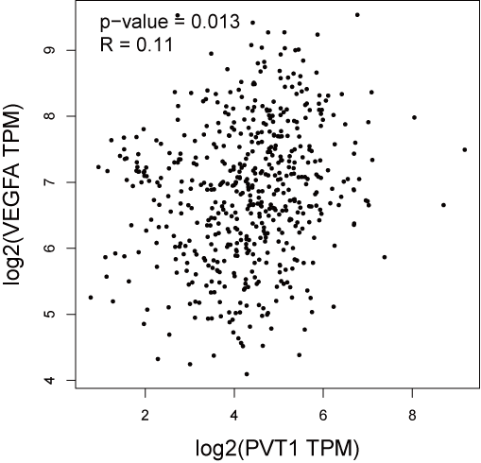


**Fig. S9** The correlation of expression between PVT1 and VEGFA was analyzed in lung cancer using the GEPIA website.


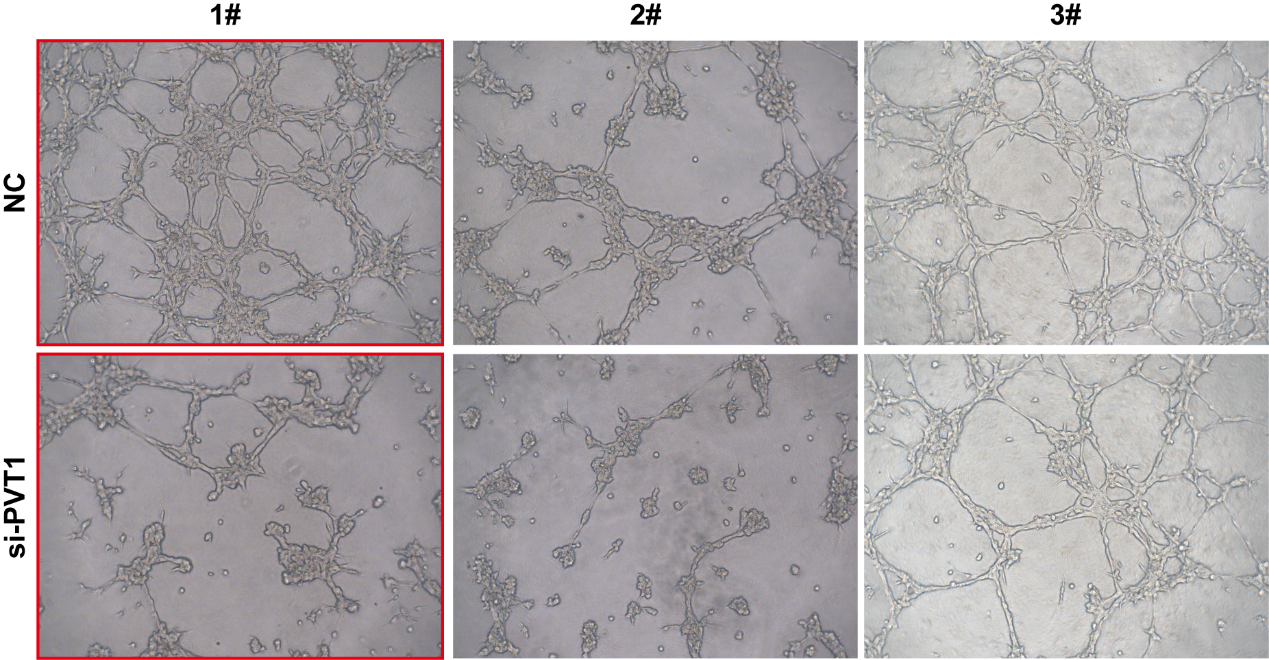


**Fig. S10** Three replicates of tube formation with conditioned medium from A549 cells transfected with si-PVT1 or NC. Red boxes represent the typical cases which are used in Fig.6.


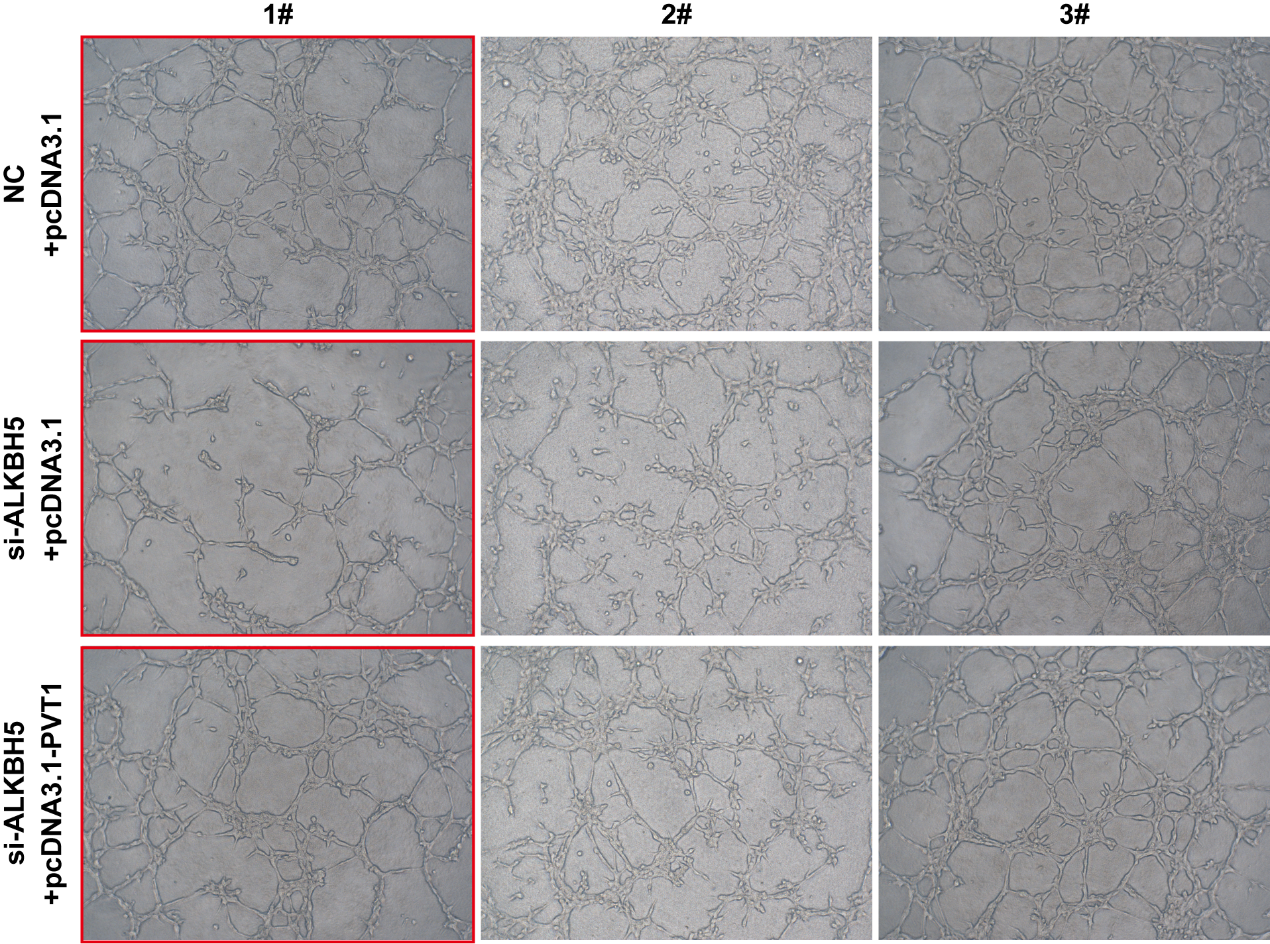


**Fig. S11** Three replicates of tube formation with conditioned medium from A549 cells transfected with si-ALKBH5 or NC and pcDNA3.1-PVT1 or pcDNA3.1. Red boxes represent the typical cases which are used in Fig.7.


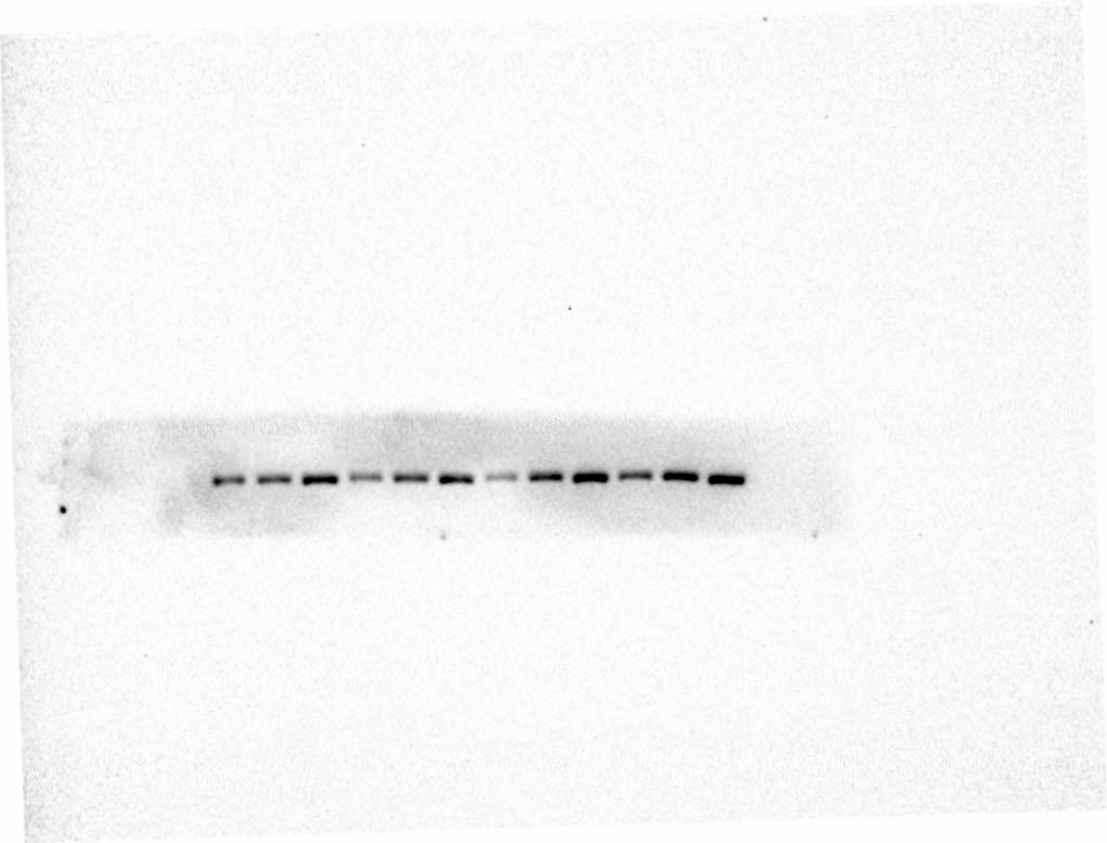


si2-ALKBH5

si1-ALKBH5

NC

**Original image for Fig 2B** Western blot of ALKBH5 antibody in A549


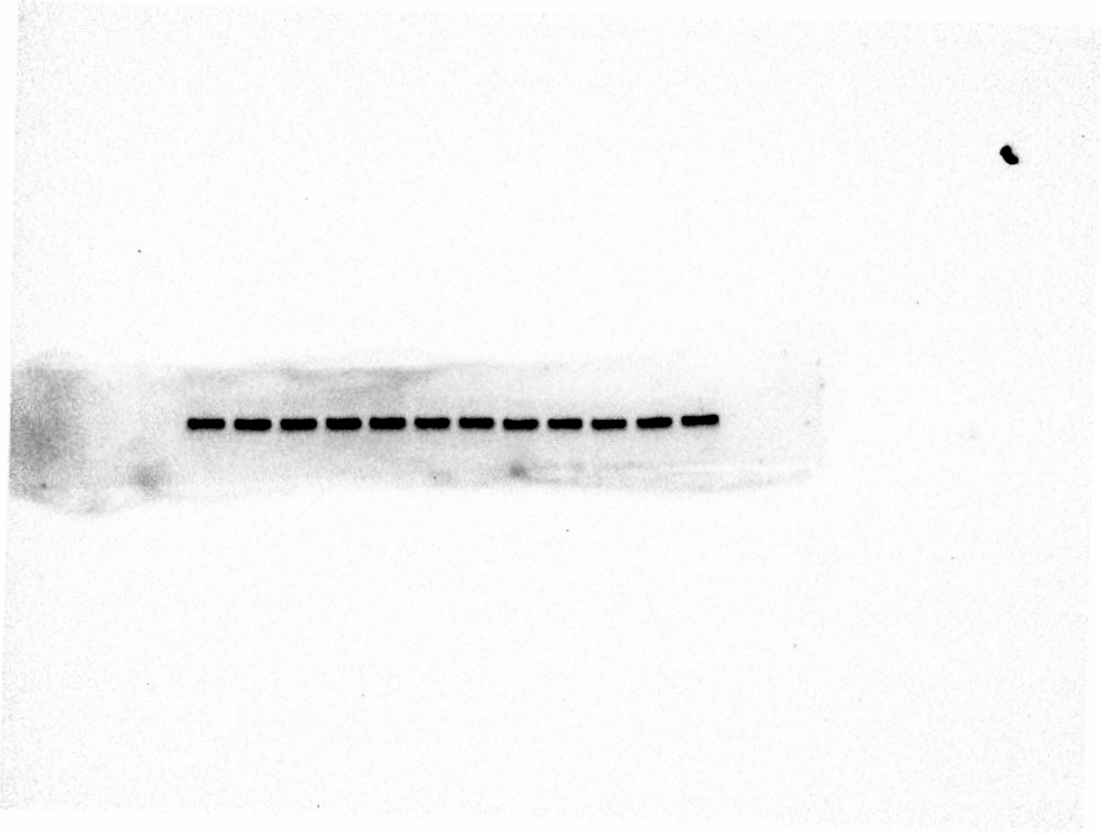


si2-ALKBH5

si1-ALKBH5

NC

**Original image for Fig 2B** Western blot of GAPDH antibody in A549


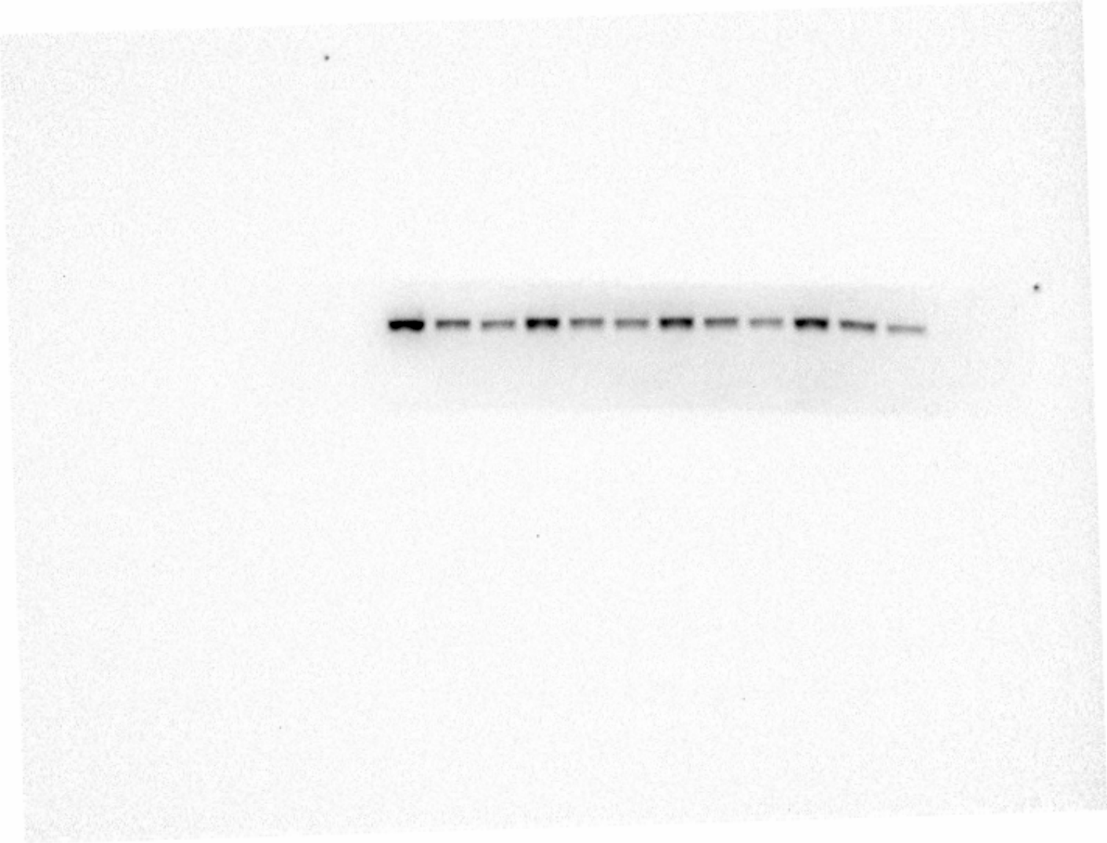


si1-ALKBH5

NC

si2-ALKBH5

**Original image for Fig 2B** Western blot of ALKBH5 antibody in H1975


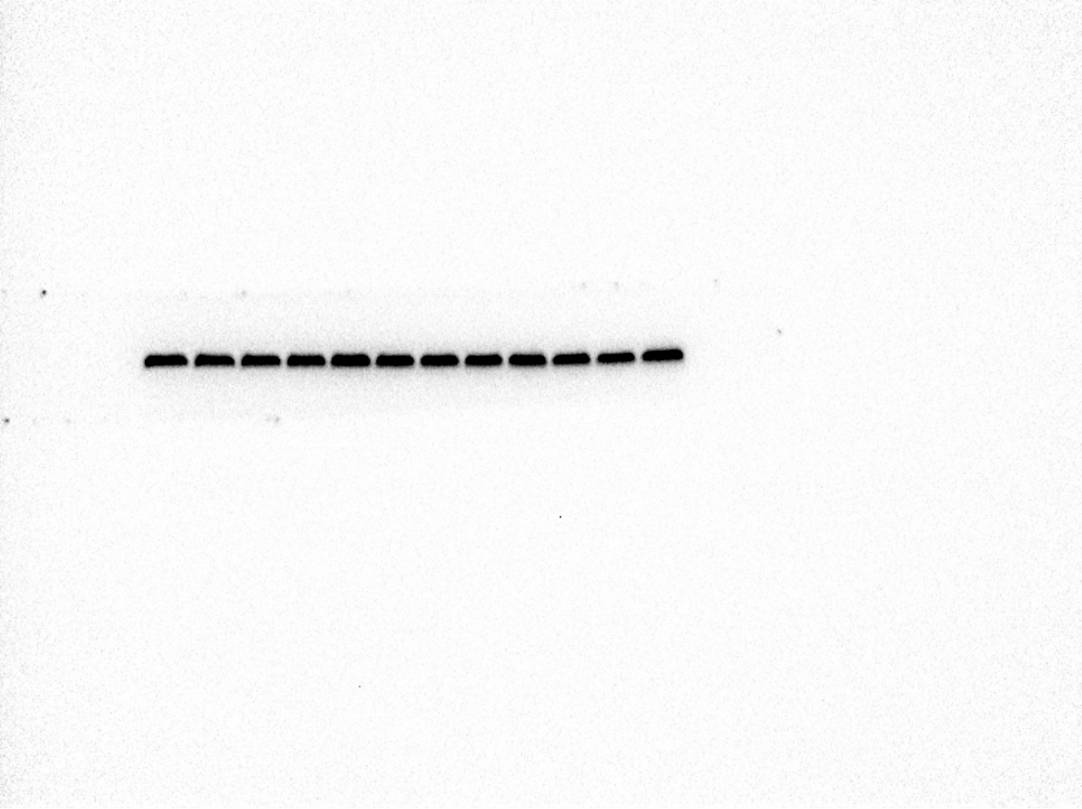


NC

si1-ALKBH5

si2-ALKBH5

**Original image for Fig 2B** Western blot of GAPDH antibody in H1975

NC

si1-ALKBH5

si2-ALKBH5

si-PVT1

NC


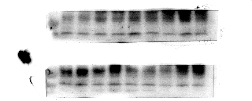


A549

H1975

NC

si-PVT1

si2-ALKBH5

NC

si1-ALKBH5

**Original image for Fig 4C 6K** Western blot of VEGFA antibody in A549 and H1975


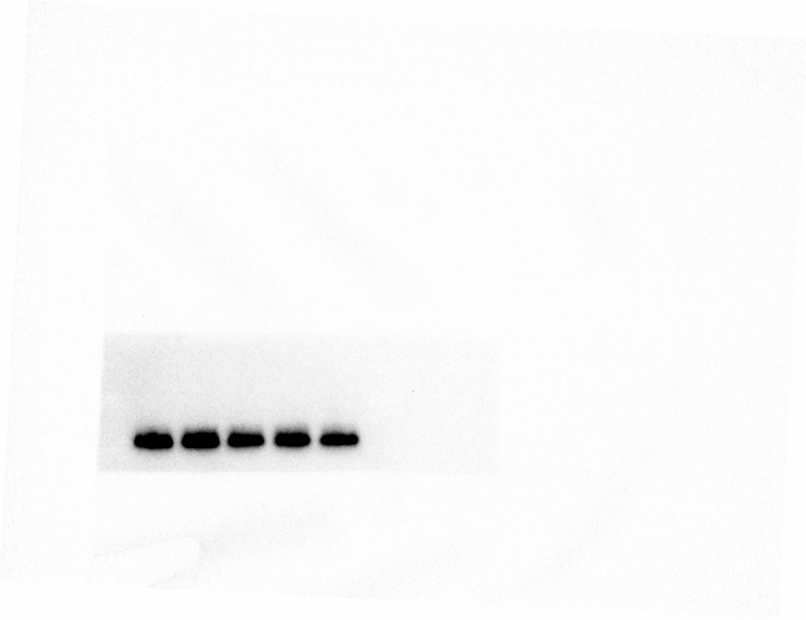


NC

si-PVT1

si1-ALKBH5

NC

si2-ALKBH5

**Original image for Fig 4C 6K** Western blot of GAPDH antibody in A549





NC

si2-ALKBH5

si-PVT1

NC

si1-ALKBH5

**Original image for Fig 4C 6K** Western blot of GAPDH antibody in H1975
